# A metagenomic investigation of the faecal RNA virome structure of asymptomatic chickens obtained from a commercial farm in Durban, KwaZulu-Natal province, South Africa

**DOI:** 10.1186/s12864-024-10517-6

**Published:** 2024-06-24

**Authors:** Vivian C. Nwokorogu, Santhosh Pillai, James E. San, Charlene Pillay, Martin M. Nyaga, Saheed Sabiu

**Affiliations:** 1 Department of Biotechnology and Food Science, Durban University of Technology, P.O. Box 1334, Durban, 4000 South Africa; 2 Next Generation Sequencing Unit, Division of Virology, Faculty of Health Sciences, University of the Free State, P.O. Box 339, Bloemfontein, 9300 South Africa; 3 Nelson Mandela School of Medicine, KwaZulu-Natal Research Innovation and Sequencing platform unit, University of KwaZulu- Natal, 719 Umbilo Road, Durban, 4001 South Africa

**Keywords:** Viral metagenomics, Faecal virome, RNA viruses, Next-generation sequencing, Poultry, Chicken, Zoonosis and, Viral diversity

## Abstract

**Background:**

Virome studies on birds, including chickens are relatively scarce, particularly from the African continent. Despite the continuous evolution of RNA viruses and severe losses recorded in poultry from seasonal viral outbreaks, the information on RNA virome composition is even scantier as a result of their highly unstable nature, genetic diversity, and difficulties associated with characterization. Also, information on factors that may modulate the occurrence of some viruses in birds is limited, particularly for domesticated birds. Viral metagenomics through advancements in sequencing technologies, has enabled the characterization of the entire virome of diverse host species using various samples.

**Methods:**

The complex RNA viral constituents present in 27 faecal samples of asymptomatic chickens from a South African farm collected at 3-time points from two independent seasons were determined, and the impact of the chicken’s age and collection season on viral abundance and diversity was further investigated. The study utilized the non-invasive faecal sampling method, mRNA viral targeted enrichment steps, a whole transcriptome amplification strategy, Illumina sequencing, and bioinformatics tools.

**Results:**

The results obtained revealed a total of 48 viral species spanning across 11 orders, 15 families and 21 genera. Viral RNA families such as *Coronaviridae, Picornaviridae, Reoviridae, Astroviridae, Caliciviridae, Picorbirnaviridae* and *Retroviridae* were abundant, among which picornaviruses, demonstrated a 100% prevalence across the three age groups (2, 4 and 7 weeks) and two seasons (summer and winter) of the 27 faecal samples investigated. A further probe into the extent of variation between the different chicken groups investigated indicated that viral diversity and abundance were significantly influenced by age (*P* = 0.01099) and season (*P* = 0.00099) between chicken groups, while there was no effect on viral shedding within samples in a group (alpha diversity) for age (*P* = 0.146) and season (*P* = 0.242).

**Conclusion:**

The presence of an exceedingly varied chicken RNA virome, encompassing avian, mammalian, fungal, and dietary-associated viruses, underscores the complexities inherent in comprehending the causation, dynamics, and interspecies transmission of RNA viruses within the investigated chicken population. Hence, chickens, even in the absence of discernible symptoms, can harbour viruses that may exhibit opportunistic, commensal, or pathogenic characteristics.

**Supplementary Information:**

The online version contains supplementary material available at 10.1186/s12864-024-10517-6.

## Introduction

Globally, chicken’s productive performance and feed conversion rate are greatly influenced and dependent on the state of health and proper functioning of its gastrointestinal tract (GIT), which is the site of nutrition, metabolism, and build-up of diverse microorganism [1]. The chicken GIT is often colonized by microorganisms like bacteria, fungi, and viruses which could be harmless, symbiotic, or pathogenic. Therefore, poor GIT health, even in the absence of a recognized disease state, can affect poultry performance, resulting in low productivity [2]. Decades of avian research, particularly microbiome studies have focused on characterizing bacteria, while utilizing the 16S rRNA gene sequencing as a powerful tool to investigate the dynamics, biological and ecological roles of the GIT microbiota in chicken. Unlike bacteria, viruses are difficult to sequence and characterize because of their lack of markers or conserved regions that can be employed for taxonomic identification, high genetic variability, and short genome lengths [3]. Although immense studies have availed valuable information about the gut microbiome, comprehensive analysis on the gut virome of chicken is still limited. Importantly, in the light of the frequently dense nature of chicken flocks and their possession of homogeneous gene traits, often result in their increased vulnerability to breakouts of viral infections. Hence, even on high precaution poultry farms, a wide range of viruses can accumulate, especially if the poultry birds are of different age groups. This is because the infections are often asymptomatic and therefore undetected allowing them to spread quickly, hence causing substantial economic losses [4]. In South Africa, nearly all bird flu viral outbreaks in poultry have been recorded in chickens and have cost the nation millions of rands, despite chicken being the most consumed source of animal protein. Notably, about 145 avian-flu outbreaks were recorded from different South African poultry farms between April 2021 and September 2023 with more than 4 million birds culled [5, 6]. Hence, it has remained crucial to critically examine the dynamics of viral pathogens that maybe implicated in underperformance, low productivity, and mortality in poultry production, particularly chickens.

Worldwide, next-generation sequencing (NGS), a high throughput sequencing method has allowed unprecedented advances in the characterization of complex microbial communities including viruses [1, 7, 8]. The NGS approach offers the combined advantages of speed, sensitivity, automation, and high-throughput deep sequencing and has successfully been used to characterize faecal microbiota of avian species including both wild [9, 10] and domestic birds [11, 12]. In addition, the revolutionization of viral metagenomics concerning epidemiological studies has allowed credible, faster, better detection and surveillance of multifunctional viruses in poultry. Majority of recent avian viral research have focused on zoonotic viral pathogens, or viruses causing significant economic losses in poultry while overlooking other viruses which also constitute the avian virome [10, 13]. Continuous viral surveillance aimed at characterizing viruses in chicken is necessary to enhance knowledge of key viral agents associated with poultry related infection. While DNA viruses such as adenoviruses [14, 15] and parvoviruses [16, 17] identified in chickens have been associated with enteritis, studies have shown that RNA viruses constitute a greater proportion of all infections caused by viruses [18, 19]. This has been attributed to the highly unstable nature of RNA viruses, and rapid mutation rates due to their error-prone replication mechanisms often leading to diverse variants and multi-species [20]. As a result of these attributed factors and their remarkable capacity to transcend species boundaries, many viruses possessing RNA genomes have emerged as noteworthy pathogens with the potential to cause widespread epidemics or even pandemics. Some notable examples of RNA viral outbreaks associated with animal origin, include severe acute respiratory syndrome coronavirus 2 (SARS-CoV 2) [21], Ebola [22], Swine flu [23], and Middle East respiratory syndrome [24]. Unfortunately, virome studies on birds including chickens are relatively scarce, particularly from the African continent, and the information on their RNA virome composition is even scantier despite the continuous evolution of RNA viruses and their associated disease outbreaks. Considering the high instability of RNA viruses, their high adaptive mechanism and quasi species emergence for instance SARS-CoV 2, raise concerns as undetected and unidentified RNA viruses may become the basis for a potential outbreak in the nearest future [3]. This study was underscored as a crucial step toward future epidemiological investigations of chicken’s faecal RNA viruses in South Africa. Importantly, the focus of the study was not on any specific pathogen (s) or disease condition(s) but rather on the entire RNA virome, hence no diseased group/chickens with defined disease condition was included. Therefore, this pilot study looked to obtain baseline data and unveil the dynamics of the faecal RNA virome of asymptomatic South African chickens.

In this study, for the first time, we conducted a comprehensive analysis to elucidate the RNA virome in the GIT of asymptomatic chickens from Durban, KwaZulu-Natal province in South Africa. Through optimized enrichment strategies, non-invasive faecal sampling methods, and bioinformatics tools, we explored the RNA virome structure of apparently healthy chickens, tracking viruses across different stages and seasons. This study provided insights into the genomes of faecal RNA viruses, their diversity, structure, and colonization in healthy chickens’ gut, while also contributing to the understanding of potential disease agents and those with potential to cross species barriers.

## Materials and methods

### Sampling design and collection

The faecal samples were obtained from a commercial poultry farm in Durban, KwaZulu-Natal, South Africa. A total of 10 chickens were used in all, five for summer and five for winter periods. Samples were collected at three different time points, namely the early (2 weeks), intermediate (4 weeks), and mature (7 weeks) developmental stages of chickens (Fig. S1). Five asymptomatic chickens were randomly pre-selected from flocks at 2 weeks of development during the summer period, July/August 2021. At the first collection time point, the selected chickens (*n* = 5) at 2 weeks were marked and kept separately from the flock in medium-sized metal cages, layered with sterile plastic wrap and sawdust. The faecal contents from each cage were obtained from individual chicken immediately as they dropped using sterile plastic bags and the chicken returned to its flock. At 4 and 7 weeks of age, the same sample collection procedure was repeated for the marked chickens (same summer chickens). During winter (December 2021 to January 2022), another five asymptomatic chickens were selected at 2 weeks, the same separation, faecal sample collection time points and collection procedure used for summer sampled chickens was followed. The demographic data of the individual samples and the 10 chickens are shown in Table S1. Notably, at age 7 weeks, only two samples were obtained from summer samples, while the remaining 3 chickens under study whose faecal samples were not obtained had been sold at the time of sample collection. This reduced the expected sample number from 30 to 27. Hence, a total of 27 faecal samples (*n* = 12 for summer) and (*n* = 15 for winter) were achieved and stored at -80 °C.

### Antibiotic pretreatment

The treatment of faecal sample with antibiotics in this study was employed as an enrichment process of selectively depleting/minimizing the interferences of non-viral nucleic acids. Pre-treatment of the faecal samples with antibiotics was done as described by Theuns et al.  [25]. A 20% chicken faecal suspension was formulated by adding 200 mg of chicken feces into 1 ml of freshly prepared phosphate-buffered saline (PBS) (Sigma Aldrich, USA) at pH 7.5 containing 1000 U/ml penicillin, 1 mg/ml streptomycin, 1 mg/ml gentamicin and 500 U/ml amphotericin B (all from Sigma-Aldrich, USA) for antibiotic treatment. The faecal suspensions were homogenized at 3000 rpm for 1 min.

### Viral RNA enrichment

Enrichment of viral particles was carried out using the standard NetoVIR protocol [26]. Following homogenization, a centrifugation step was done at 17,000 *g* for 3 min, prior to filtration. The only modification to this method was in the filtration stage, simultaneously carried out in two steps, using a 0.8 μm syringe filter (Sigma-Aldrich, USA) and thereafter with a 0.45 μm pore size (Merck, Millipore) to filter off largely sized nuclei, mostly those pertaining to bacterial cells. The resulting filtrate obtained from each sample was subjected to nuclease treatment using a cocktail of degrading enzymes; 2 µl of 25 U/µl benzonase nuclease (Sigma-Aldrich, USA) and 100 U/µl micrococcal nucleases (Thermo Scientific, Massachusetts, USA) combined with 7 µl of freshly prepared buffer containing 1 M Tris buffer (Merck, Germany), and 30 mM MgCl_2_ (Sigma Aldrich, USA) and 100 mM CaCl_2_ (Sigma Aldrich, Missouri, USA), pH 8.0, incubated for 2 h at 37 °C to destroy the naked free-floating nucleic acids. The nuclease enzyme was inactivated with 0.5 M EDTA. Total viral RNA was extracted using QIAamp viral RNA mini kit (Qiagen, Hilden, Germany) following the manufacturer’s instructions, without the use of carrier RNA. To deplete viruses with DNA genomes, DNase treatment was performed in 50 µl reaction mix using the DNase I M0303S kit (New England Biolabs), while column purification and concentration was carried out with the RNeasy Plus Micro Kit (Qiagen Hilden, Germany) according to their manufacturer’s guidelines. To further enrich for viral RNA, the host ribosomal RNA (rRNA) depletion was achieved using the NEBNext ribosomal RNA depletion (Human/Rat/Mouse) kit (New England Biolabs, Massachusetts, USA) following the manufacturer guidelines. All viral RNA quantifications were done using a highly sensitive RNA-specific fluorometric method on Qubit 3.0, with Qubit HS RNA reagent (Life Technologies, USA). Four negative controls were incorporated at different stages to monitor kitome contamination, and their details are described in TableS2.

### Library preparation and sequencing

Prior to library preparation, cDNA synthesis and whole transcriptome amplification (WTA) of the purified ribosomal depleted RNA samples was done as described by [27]. This WTA approach involves a first step of reverse transcription of RNA into complementary DNA, flanking the primer ligation sites, and a final amplification step of the cDNA library independent of poly A-tailing and oligo d(t) priming. The libraries of all samples were individually prepared using the QIASeq FX Single Cell RNA Library Preparation Kit (Qiagen, Hilden, Germany) following the manufacturer’s guidelines. The concentrations of the resulting cDNA libraries were determined fluorometrically, and their fragment sizes assessed using the Agilent 2100 Bioanalyzer (Agilent Technologies, USA). Additional information regarding the chicken faecal samples collected are found in the supplementary data, including the quantitative data of individual chicken faecal samples at key enrichment steps (Table S3 and S4). The constructed cDNA libraries were sequenced on a MiSeq platform (Illumina, San Diego) (151 × 2 cycles using a V3 600 cycle kit) alongside four control samples at the next-generation sequencing unit of the University of the Free State, Bloemfontein, South Africa.

### Bioinformatics analyses

All processing of the resulting raw paired-end reads retrieved as FASTQ files was carried out using Genome Detective (GD) virus tool- panviral version 2.52 [28]. Briefly, this automated high performance online pipeline for viral detection incorporates Trimmomatic [29] and FastQC integrated FASTQ [30] for the removal of low quality, uninformative reads, as well as in-depth quality control. Candidate viral reads are then identified using the protein-based alignment method DIAMOND [31] against a subset of the Swissprot UniRef90 protein database to improve sensitivity and speed. The database contains more than 490,000 representative clusters of proteins linked to taxonomy IDs and is constantly updated. By performing the primary search at amino acid (aa) level, GD can accurately classify reads that have diverged from the references on nucleotide (nt) level. Short reads representing the same viral species are separated into separate groups, or buckets. Each bucket contains all reads from a single taxonomy ID based on the Least Common Ancestor (LCA) of hits identified by DIAMOND score. Each bucket is then de novo assembled separately using metaSPAdes [32] for paired-end reads and scaffolds classified. BLASTx and BLASTn was used to search for reference sequences against the NCBI RefSeq virus databases to confirm that the viral taxonomic ID of the resulting *de novo* contigs produced agrees with the bucket and identify candidate reference sequences of the resulting *de novo* contigs produced against NCBI RefSeq virus database. The GD pipeline then combines the total blastn score and blastx score results for every detected contig to simultaneously take into account amino acid and nucleotide similarity and selects five best scoring references for each contig to be used during alignment. Finally, the contigs for each individual species are stitched together using Advanced Genome Aligner (AGA) [33] to produce the consensus sequence. In this study, viruses were assumed to be contaminants due to index-hopping from another library if the total read count was less than 0.1% of the most abundant read count of the same virus(es) and cross-checked with a pre-existing compilation of viral contaminants [10, 34]. Also, viruses detected in the negative control libraries of reagent mix incorporated at different stages, and/or belonging to the same clades as those detected in blank libraries was presumed to have originated from contamination that is most likely linked to laboratory reagents [35, 36]. Hence, those viruses were eliminated from the chicken libraries and excluded from all further downstream investigations.

### Viral diversity and abundance across age groups and seasons

To adjust for variations in read depth across libraries, the measure of abundance was expressed as the read count of reads per million, achieved by dividing the read count by the total number of reads in the library and then multiplying the quotient by one million. The effects of age and seasons on chicken gut virome diversities was investigated using ecological diversity metrics (alpha and beta diversity). While the alpha diversity measures explored were Sobs (number of observed genera), Shannon-Weiner and Simpson index, the beta diversity was determined using the abundances of the principal coordinate analysis (PCoA) based on the Bray-Curtis dissimilarity index [37] and Jaccard presence of absence theorem [38, 39]. The statistical evaluation of the alpha and beta diversities was achieved using Kruskal-Wallis’s rank sum and Adonis test using the Bray-Curtis beta diversity results computed with 1000 replicates, respectively on sample sequences from each sample as well as each group.

### Data visualization

The data visualizations and statistical analyses employed in the present study were mainly performed using the R Software (version 4.3.0)). The abundance data generated as reads and taxonomy tables were used as input files for further visualization on R, while utilizing a suite of packages including Vegan [40], ggplot2 [41], cowplot [42], pheatmap [43], and tidyverse [44]. Determination of differences between the sample groups observed in relation to chicken age and seasons was explored. Linear models and permutation analysis of variance were employed to test for these differences in richness and diversity indices across groups (age and season).

### Phylogenetic analysis

The analysis of genetic phylogenies was conducted with only viruses having full-length genomes with ≥ 90% of its total genome coverage or partial genomes that have complete RNA-dependent RNA polymerase (RdRp) genes. Multiple sequence alignment was performed in the MUltiple Sequence Comparison by Log-Expectation (MUSCLE) software [45] comparing them with global sequences retrieved from GenBank and best hit results of BLAST searches with an expected e-value threshold 1 × 10^− 3^. The maximum likelihood method was applied to infer phylogenies using the software Molecular Evolutionary Genetics Analysis (MEGA v 11.0) [46]. The resulting phylogenies were visualized and annotated using FigTree v1. 3.1 [47].

### Ethics approval

The ethical approval of this study was obtained from the Animal Research Ethics Committee (AREC) of the University of KwaZulu-Natal with the protocol Ethics Number, AREC 012/020. In addition, in compliance with Sect. 20 of the Animal Disease Act (Act No 35 of 1984), a research permit was obtained from the South African Department of Agriculture, Land Reform and Rural Development, with the reference no 12/1/5/4 (1511AC). Furthermore, for faecal samples, a provincial no restriction permit was obtained from the KwaZulu-Natal Department of Agricultural and Rural Development.

## Results and discussion

### Overview of viral metagenomic analysis

**Fig. 1:**
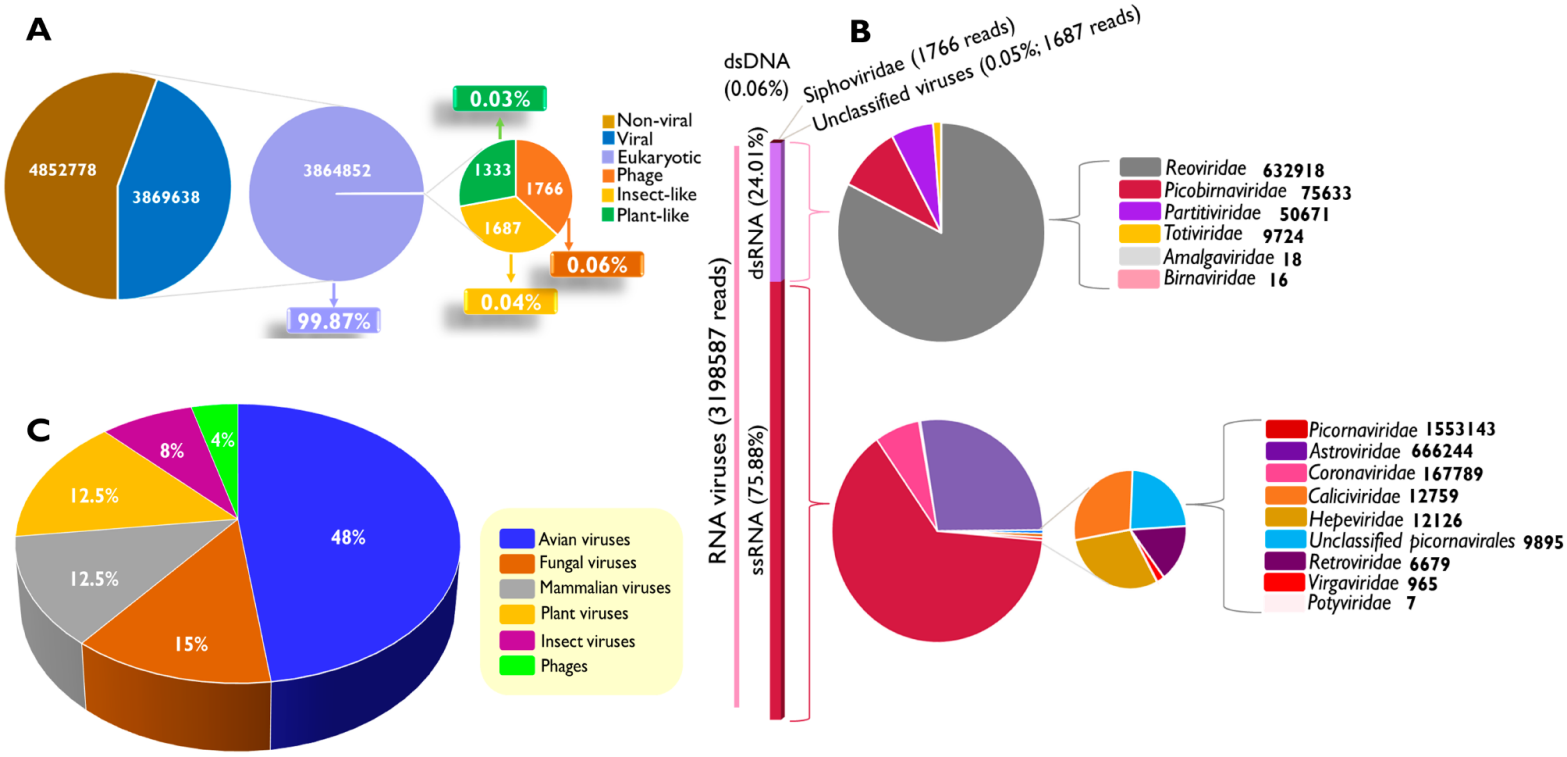
The distribution of sequence reads obtained from metagenomic analysis chicken faecal samples. **(A)**. The distribution of viral and non-viral sequence reads obtained from studied chickens (first pie), the middle pie depicts the prevalence of eukaryotic viral reads, from which smaller proportion of viral reads (%) were illustrated in the last pie. **(B)** The composition and diversity of viruses identified in chickens based on reads. The left bar denotes the numbers of assigned viral reads by genome type in brackets, RNA viruses (ssRNA and dsRNA). The pie charts show the composition of viral families identified by total number contig of its respective members in bold faces. **(C)** A Pie chart of the percentage host distribution of 48 different viral species identified from the study as correlated by the corresponding color in legend on the right

In this study, a total of 9,429276 sequence reads, were generated from 27 cDNA libraries of chicken faecal samples and a notable Phred score of 38 achieved across all samples using the Illumina Miseq. The unassigned reads accounted for 5,559638 reads; a larger proportion of the reads generated in this study. It was extrapolated that slightly above half of the generated reads were non-viral reads, while viral reads with hits to eukaryotic viruses far outweighed those aligning to phages (0.06%), insects (0.04%) and plant (0.03%) viruses with a 99.88% prevalence (Fig. 1A). Despite the lower viral reads, from a total of 8,722,416 reads after quality check, 3,869638 were assembled into 4328 viral contigs. Higher dominance of non-viral read to viral reads has been reported in metagenomics studies, including domestic birds [48, 49]. Among 48 viral species identified, 15 viral species, majorly comprising avian viruses had complete and near complete genomes (> 95%) (Table 1). In addition, other viral species with less than 95% genome, such as *Quail picornavirus QPV1/HUN/2010*, *Gallivirus A* and *Megrivirus C*, had full length RdRp or polyprotein gene except *Infectious bursal disease virus* from 7W5 with only 23% genome coverage of its segment B.

**Table 1:** Nearly complete genomes (> 95%) generated from apparently healthy chicken faecal samples in the present study

Genome type	Virus species	Percentage genome coverage
Unsegmented viruses	*Avian coronavirus*	99.3% (2S4), 99.0% (2S5)
	*Avihepevirus magniiceur*	98.8% (7W4)
	*Bavaria virus*	99.3% (2S5), 98.5% (2S4)
	*Chicken astrovirus*	99.9% (2W4, 7W2, 7W3), 98.8% (7W1), 99.0% (7W4)
	*Megrivirus C*	100% (7W5), 99.8% (2W3), 98.7% (2W2), 97.7% (2W5), 97.6% (4W5)
	*Orivirus A*	96.1% (2W2)
	*Sicinivirus A*	96.0% (7W2)
Segmented viruses	*Rotavirus F*	2W1: 11 segments; VP1(99.5%), VP2(100.0%), VP3(100.0%), VP4(100.0%), VP6 (97.7%), VP7(100.0%), NSP1(99.3%), NSP2(100.0%), NSP3(100.0%), NSP4(100.0%), NSP5(96.8%)
		2W2: 10 segments; VP1(98.9%), VP2(97.7%), VP3(100.0%), VP4 (98.2%), VP6(100.0%), VP7(100), NSP2(100), NSP3(100), NSP4(100.0%), NSP5(100.0%)
	*Rotavirus G*	2S1: 8 segments: VP1(99.0%), VP2(98.5%), VP3(98.6%), VP7(96.8%), NSP2(100.0%), NSP3 (100.0%), NSP4(100.0%), NSP5(100.0%)
		2S5: 2 segments; VP3(98.2%), VP6(100.0%),
		2W3: 10 segments; VP1(99.7%), VP2(98.6%), VP3(98.4%), VP4(100.0%), VP6(100.0%), VP7(96.0%), NSP1(100.0%), NSP2(99.0%), NSP4(100.0%), NSP5(98.4%)
		4S1: 5 segments; VP3(98.6%), VP6(100.0%), VP7(96.4%), NSP1(100.0%), NSP2(99.7%)
		4S2: 9 segments; VP1(99.3%), VP2(97.9%), VP3(98.6%), VP6(100.0%), VP7(99.6%), NSP2(100.0%), NSP3(100.0%), NSP4(100.0%), NSP5(100.0%)
		4S4: 2 segments; VP3(97.7%), VP6(99.7%)
		4S5: 6 segments; VP1(99.5%), VP2(97.9%), VP3(100.0%), VP6(100.0%), NSP1(100.0%), NSP5(99.5%)
Segmented viruses	*Avian orthoreovirus*	2W1: 2 segments; L2(98.7%), L3(99.0%)
		2W4: 2 segments; L2(99.7%), M2(99.4%)
	*Chicken Picorbirnavirus*	Only 1 segment; RNA 2; 99% (2S2), 99.6% (4S5): 97.8%(4S2), 97.4% (2S5).
	*Porcine picorbirnavirus*	Only 1 segment; segment S; 97.6%(2S1)
	*Picobirnavirus dog/KNA/2015*	Only 1 segment: segment 2; 99.6% (4W1), 98.6% (4S5), 99.4% (7W2), 98.8% (4W5)
	*Otarine picorbinavirus*	98.3% (2S1), 98.2% (2S2), 98.5% (2S5).
	*Aspergillius fumigatus Partitivirus*	Only 1 segment: segment 1; 97.8% (4W2)

### Chicken faecal RNA virome

The taxonomic analysis of the 3,869638 viral reads obtained revealed a total of 48 different viral species spanning across 15 viral families, 21 genera and some unclassified viruses. Based on genome type, a total of 43 viral species pertaining to RNA viruses, 2 DNA tail phages (from *Siphoviridae* family) and 3 unclassified viruses (Fig. 1B). It was observed that within the RNA viral species obtained, 25 were dsRNA viruses (75.88%) while 18 were ssRNA viruses (24.01%), with total viral read percentages 75.88% and 24.01% excluding phage and unclassified viruses (Fig. 1B). The two dsDNA tail phages (*Escherichia virus DE3* and *Lambdavirus lvO276*) recovered occurred only in one winter sample each, 7W3 and 2W4 respectively. Notably, previous studies on chicken gut virome reported the presence of tail phages from families *Myoviridae Podoviridae* and *Siphoviridae* in American [2] and UK [48] chickens, despite the former employing DNA removal procedures. Also, the absence of avian DNA viruses associated with the gut of chicken could mean that the enrichment and DNase treatment procedures undertaken were effective. The number of RNA viruses identified showed high RNA viral diversity.

The classification of identified viruses based on their established host specificity, exhibited a remarkably broad host range with the avian viruses having the highest prevalence of 48% [23] while mammalian and plant viruses accounted for 12.5% each (Fig. 1C). Phages were the least abundant, constituting only 4% of the host-virus population while Insect viruses accounted for 8% of the population (Fig. 1C), in addition to 3 Unclassified ShiM-2016 viruses (Table S5). The considerably high prevalence of avian viruses can be attributed to the targeted enrichment strategy utilized which significantly reduced the presence of non-viral genomes.

**Fig. 2:**
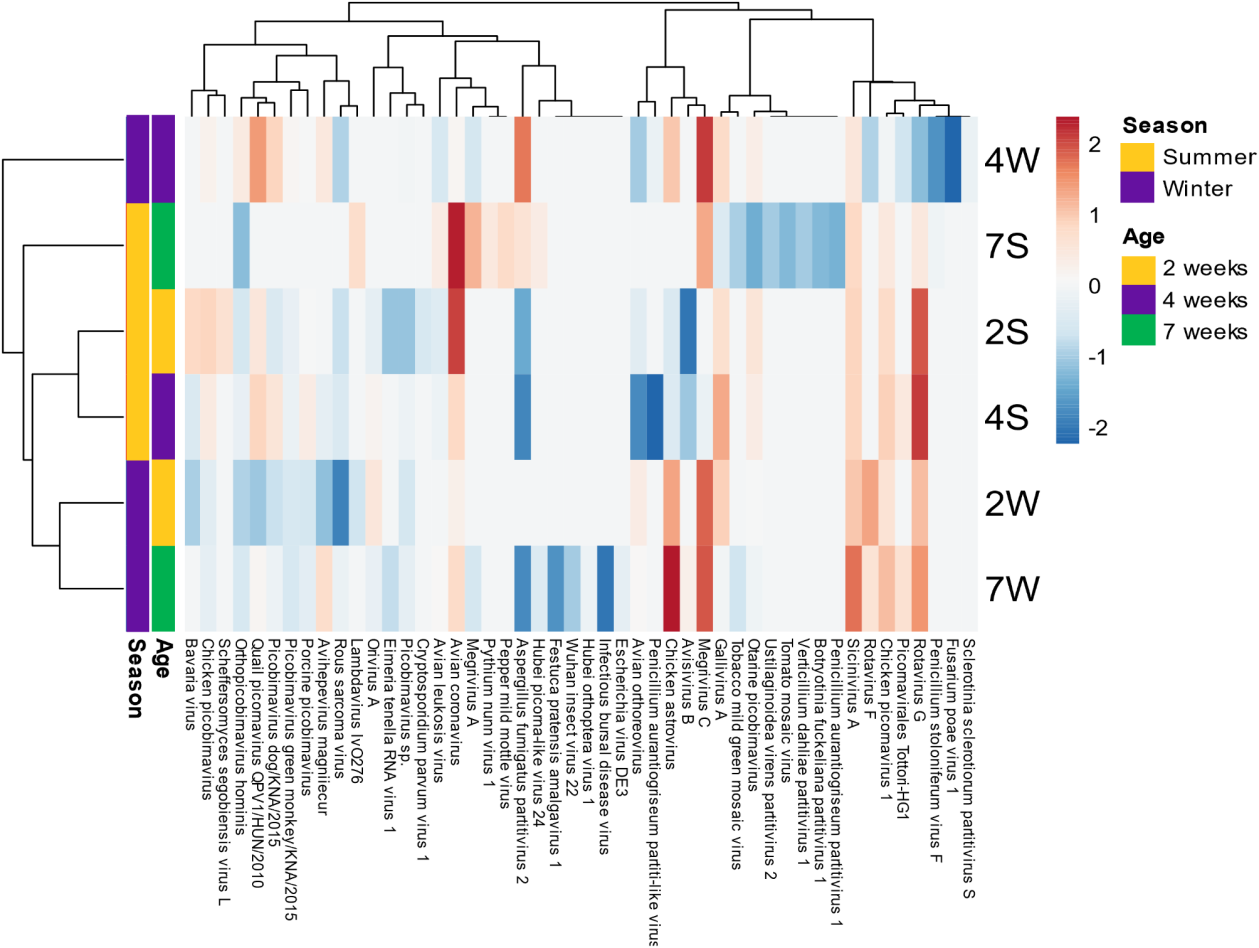
The overall read abundance of viral species from 27 chicken study samples across 2-, 4- and 7-weeks age groups categorized by summer and winter sample collection time points

Members of seven viral families, *Picornaviridae, Reoviridae, Astroviridae, Coronaviridae, Caliciviridae, Picobirnaviridae* and *Retroviridae* were majorly to found across samples. However, it is noteworthy that the most abundant viral families are *Picornaviridae, Astroviridae*, *Reoviridae* and *Coronaviridae* with 1,550559, 666,244, 632,918 and 167,789 viral reads, respectively (Fig. 2; Table S5). The recovery of these viral families across samples may imply that they are more often detected from chickens. Importantly, the occurrence of these viral families in this study is consistent with previous reports on metagenomic investigation of chicken gut virome [1, 2, 11, 48]. These studies have commonly identified all the viral families with exception to only few studies reporting member of the *Retroviridae* mainly in diseased chickens presented with Rou sarcoma or avian leukosis lesions [50, 51]. Notably, the observed most abundant viral families (*Picornaviridae*, *Astroviridae*, *Reoviridae* and *Coronaviridae*) have been reported from similar studies on chicken virome from Netherlands [49], Brazil [11], UK [48], and Switzerland [52]. In addition, tracheal virome and respiratory studies on broiler chicken reported only two of these families, Picornaviridae and Coronaviridae in high abundance [12, 53].

At species level, *Sicinivirus A* and *Avian coronavirus* were found be abundant across all sample group (Fig. 2). In addition, viral specie read abundance for each individual sample showed that many viral species from *Picornavirida*e family were found in nearly all samples (Fig S2). Based on the occurrence of viral species across the 27 samples, *Picornaviruses*, *Sicinivirus A* and *Gallivirus A* were highest, occurring in 27 and 26 samples respectively while nearly fungal and plant viruses had low occurrence across samples except the *Infectious bursal disease virus* (Fig S3A). At family level, *Picornavirida*e was 100% prevalent across the 27 samples followed by *Reoviridae*, occurring in 24 (88.9%) samples (Fig S3B). Recent studies have identified a variety of picornavirus genera in seemingly healthy bird species from different avian orders particularly, the *Galliformes* [12], *Anseriformes* [10], and *Charadriiformes* [9]. Hence, it could be deduced that picornaviruses are normal flora of the chicken’s gut since studies have reported them to be prevalent in healthy and/or diseased states [1, 11, 48, 52] and across different developmental stages [1, 12, 49].

Overall, the data obtained from this metagenomic study of asymptomatic South African chickens reveals high faecal RNA viral diversity that may be circulating in poultry farms. In addition, it could be highlighted that even in the absence of an observable overt disease condition, the gut RNA virome of chicken are highly diversified.

### Identification and characterization of avian RNA viruses from asymptomatic chicken faeces

A total of 3,129,023 viral reads were assigned to avian RNA viruses encompassing 11 viral families (Table S6). This includes three dsRNA avian viral families (*Reoviridae, Birnaviridae* and *Picobirnaviridae*) comprising five viral species, 6 distinct ssRNA viral families with 14 viral species and unclassified ssRNA viruses (Table S6). The ssRNA viral families (*Coronaviridae, Picornaviridae*, *Caliciviridae*, *Astroviridae*, *Retroviridae*, and *Hepeviridae*) yielded about 77% (2,418,740) of the entire reads across all families including non-avian viral reads (Table S6).

#### *Reoviridae*

**Fig. 3:**
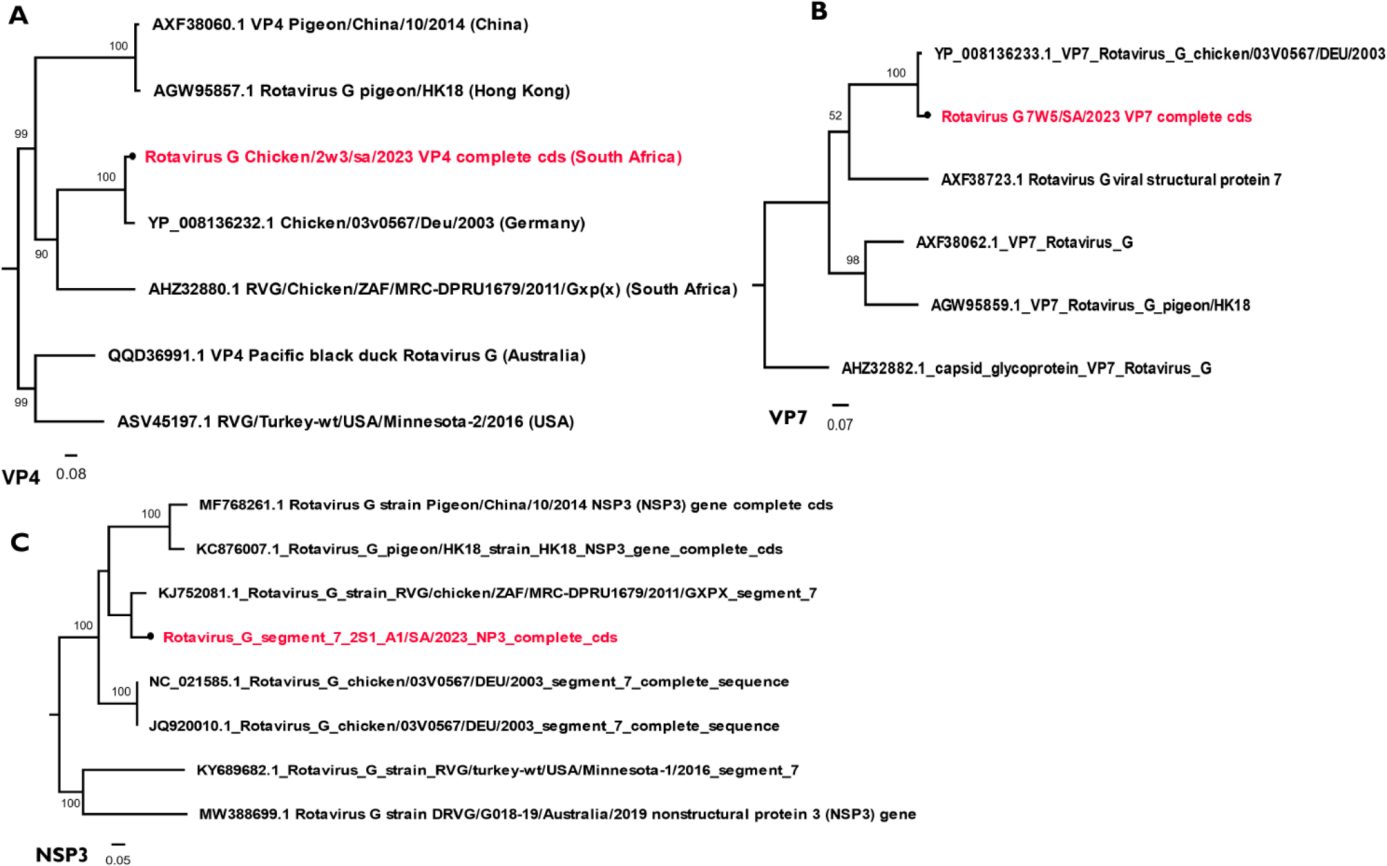
Phylogenetic analysis of *Rotavirus G* virus segments, using the maximum likelihood, Tamura 3 parameter model. The trees were midpoint rooted for clarity and the branch length support was estimated using 1000 bootstrap replicates and virus labels in red fonts denotes the viruses identified from chicken faecal sample in this study. **A**. Phylogeny of theVP4 segment of *Rotavirus G*. **B**. Phylogeny of the VP7 segment of *Rotavirus G.***C**. The phylogeny of the NSP3 segment of *Rotavirus G*

**Fig. 4:**
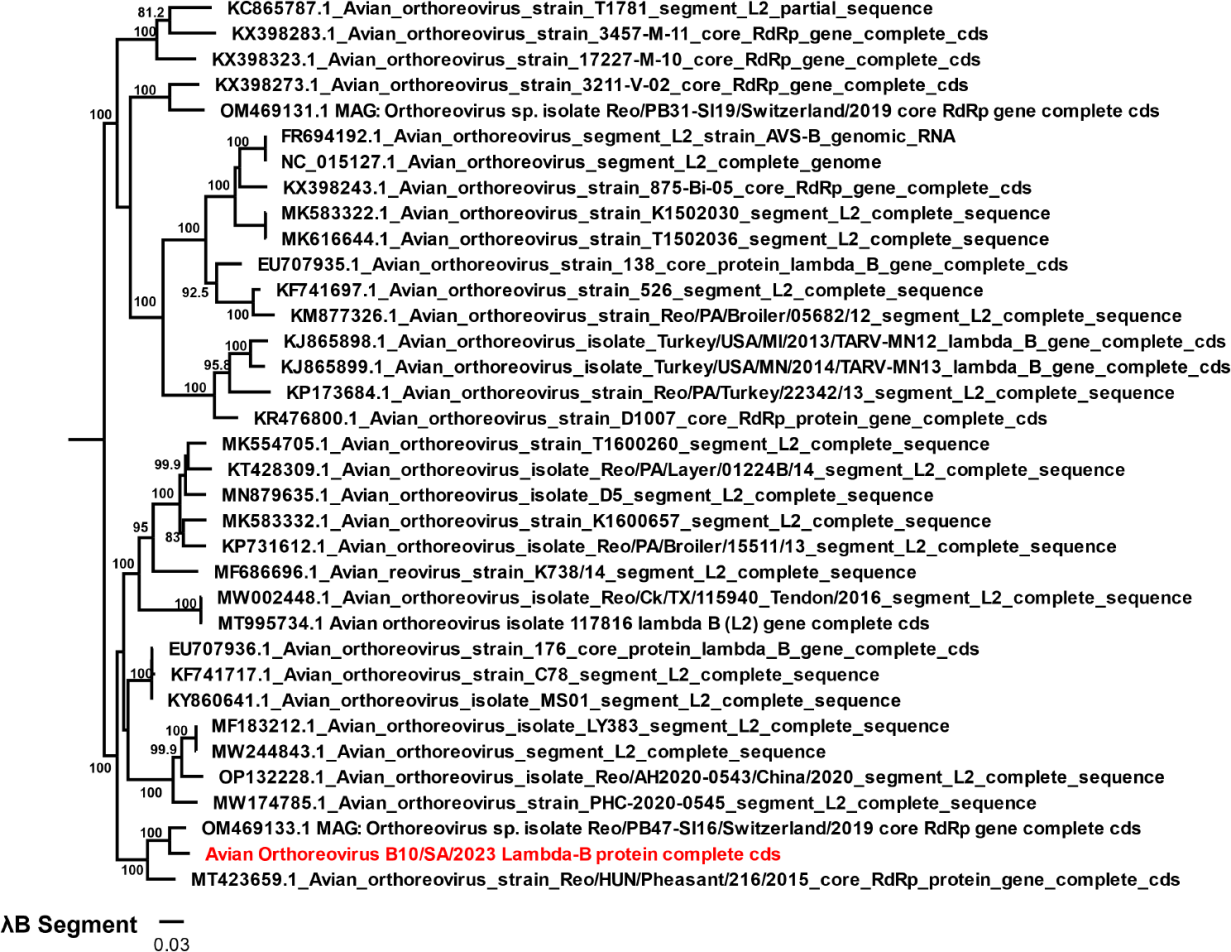
The maximum likelihood phylogenetic analysis of *Avian orthoreovirus* using the Lambda-B (RdRp). The tree was midpoint rooted for clarity and the branch length support was estimated using 1000 bootstrap replicates with the Tamura 3 model, while *Avian orthoreovirus* identified in this study is depicted in red fonts. The viruses in blue font had the highest identity with the identified virus

Three reoviral species were obtained in this study from two genera, *Orthoreoviru*s and *Rotavirus*. The two rotaviral species obtained were *Rotavirus F* and *Rotavirus G* with 205,450 and 413,022 reads, respectively (Table S6). The nucleotide and protein-coding sequences of all eleven segments of *Rotavirus F* and *Rotavirus G* were obtained from 2W1, 2W2, and 2W2 for the former, and 2W3 and 7W5 for the latter. Seasonally, *Rotavirus G* prevailed in both seasons, while *Rotavirus F* only occurred in winter samples only which may imply a seasonal emergence of *Rotavirus F* in for the studied chickens. The phylogenetic result of *Rotavirus G*, VP4 segment with 100% genome coverage from sample 2W3 and VP7 segment from 7W5 showed that the virus is most similar to the VP4 of strain (YP_008136232.1) (Fig. 3A) and VP7 of strain (YP_008136233.1) (Fig. 3B) of German chicken/03V0567/DEU/2003, respectively. It was also observed that it clustered distinctively as a sister clade with the RVG/chicken/ZAF/MRC-DPRU1679/2011/Gxp strain previously isolated from South Africa chicken from the Limpopo province (Fig. 3C) while the VP4 segment of Rotavirus F from 2W1, was most related to *Rotavirus sp.* from China (UHS71878.1) (Fig S4). The *Orthoreovirus* had partial and nearly complete coding gene sequences of only 7 segments (L1, L2, L3, M1, M2, S3, and S4), excluding the S1 segment coding for the sigma C protein. The segments include L1 (95%), L2 (98.7%), L3 (99.0%) and M2 (87.5%) from 2W1, 2W2 had only segment L2 (99.7%) and M2 (99.4%), while 2W4 had L1 (93.3%), L2 (99.7%), M1 (94.4), M2 (99.4), M3 (90.4%), S3 (82.6%) and S4 (91.9%). Phylogenetic result of nearly complete *Lamda* B of *Avian Orthoreovirus* showed highest relatedness to isolate Reo/PB47I16/Switzerlamd/2019 (Fig. 4). Overall, the higher viral abundance of *Avian Orthoreovirus* and *Rotavirus G* at both 2 summer and winter samples 2 weeks samples and *Rotavirus F* at week 2 winter samples may suggest high susceptibility of juvenile chickens to reoviral infections particularly rotaviruses, which are listed among the major causes of death in young chickens. Recent studies have demonstrated that younger chicks are more highly susceptible to reoviral infections, particularly *Rotavirus G* and *F* [9, 54]. In addition, studies have attributed *Avian Orthoreovirus* to arthritis and tenosynovitis [55, 56] while *Rotavirus G* and *F* have been associated with acute gastroenteritis in poultry [11, 52, 54]. Although, a minimal rate of enteritis is expected for large commercial chicken flocks, the chickens in this study were asymptomatic, showing no signs of any disease conditions as confirmed by the poultry farmers through their veterinary experts. Currently, there is limited information on *Avian orthoreoviruses, Rotavirus G*, and *F* from South Africa for more analysis and comparison, and this may imply that the true diversity of reoviruses including the subtypes circulating in South African chickens may not have been fully unravelled.

#### *Birnaviridae*

In this study, *Infectious bursal disease virus or Gumboro disease virus* segment B was identified with 4 viral contigs and a total of 626 nucleotides from only one sample, a 7-week winter (7W5) sample. This virus VP1 gene shared it highest nt identity of 95% with a novel variant of *Infectious bursal disease virus* genotype A2dB1b (OR791872.1) isolated from Egyptian chickens. It also a had 90% and 94% nt and aa identity with the RdRp (VP1) of the European “very virulent strain” isolate “UK661” of *Infectious bursal disease virus* (NC_004179.1) isolated from chicken. The occurrence of this *Avibirnavirus* is surprising and of great concern especially since the studied chickens’ received vaccine against *Gumboro disease* virus before 2 weeks of age. Nevertheless, since this virus was detected from only one sample (7W5), it could be speculated that it may be an instance of missed vaccination for that chicken. Further phylogenetic analysis of this virus could not be achieved as result of very low genome coverage (23%) of the recovered segment B. Also, the administered vaccine Nobilis® Gumboro D78 was not available across all GenBank databases for comparison with the 7W5 identified from this study, hence we could not confirm if it was a vaccine strain. However, recent studies have reported *Infectious bursal disease virus* to be higher in birds of 3–7 weeks of age, often causing bursal lesions and immune suppression that could result to susceptibility to other secondary infections, which may lead to the death in older birds while infections in younger birds may be mild [57, 58].

#### *Picobirnaviridae*

Chicken picobirnaviruses in addition to other mammalian picobirnavirus types identified from this study are still under unclassified isolates by the ICTV nomenclature. *Chicken picorbirnavirus* had a total of 2061 reads from nearly all sample age groups and seasons except 5 samples (7S2, 2W4, 2W5, 7W4, and 7W5). However, only the segment RNA 2 which is the polymerase gene were obtained across samples with 99.9%, 99.6%, and 97.8% gene segment coverage from samples 2S2, 4S5 and 4S1, respectively (Table S1). Further analysis of the RdRp gene (99.9%) of *Chicken picobirnavirus* from 2S2 showed 81.3% highest Identity with putative RdRp of *Picorbirnavirus sp* isolate 2282-K141-180086 (USE07851.1) isolated from Chinese chicken faeces (Fig S5).

#### *Coronaviridae*

**Fig. 5:**
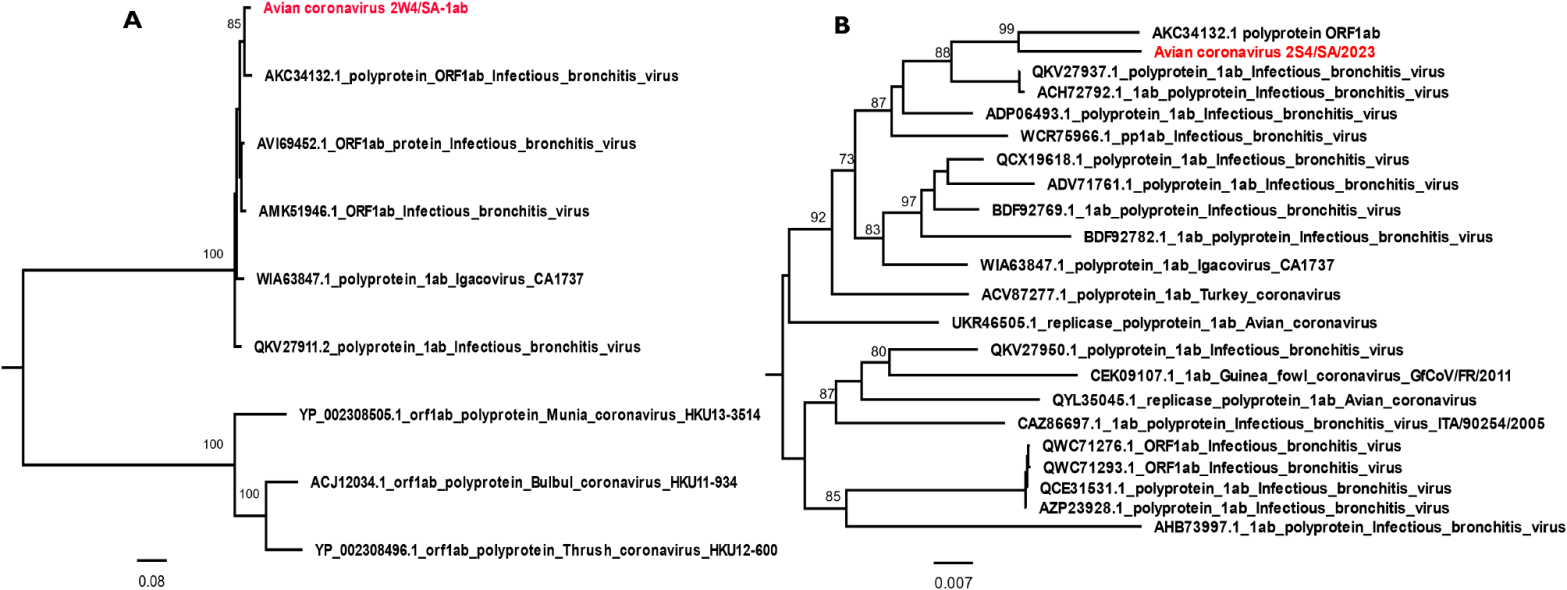
Phylogenetic analysis of *Avian coronavirus*, ORF1ab and RdRp NSP12 using the maximum likelihood, Jone-Taylor-Thornton model. The trees were midpoint rooted for clarity and the branch length support was estimated using 1000 bootstrap replicates and virus label in red fonts denotes the viruses identified from chicken faecal sample in this study. **(A)** Phylogeny of the ORF1ab segment from sample 2W4. **(B)** Phylogeny of the 1ab gene RdRP (NSP12) from sample 2S4

*Avian coronavirus* or *Infectious bronchitis virus* was obtained from this study accruing about 167,789 reads, across the 3 ages groups and two seasons. This virus demonstrated a 74% prevalence occurring in the about 20 samples out of 27 samples (Fig S3b). Nearly complete genomes of *Avian coronavirus* were recovered from samples 2S4 and 2S5 with 99.3% and 99.0% nucleotide sequence coverage, respectively. Notably, complete coding sequence (CDS) and 100% sequence coverage of the OR1ab polyprotein, NSP1 to NSP16 proteins, 3a, 3b, 5a, and 5b proteins including the spike, envelope, membrane, and hypothetical proteins were recovered from sample 2S4. Analysis of the ORF1ab polyprotein showed best hit with 95% identity to QX-like Infectious bronchitis virus isolate ck/ZA/3665/11 from South Africa (AKC34132.1) (Fig. 5A). Similarly, phylogeny result using the RdRp gene (NSP12) demonstrated a distinct cluster with the same ck/ZA/3665/11 South African strain as a sister clade to strain H120 and isolate ZJ971 of *Infectious bronchitis virus* from UK (QKV27937.1) and Chinese (ACH72792.1) chickens (Fig. 5B). The studied chickens were vaccinated against *Infectious bronchitis virus* 4–91 serotype using Nobilis® IB 4–91 and importantly presented no observable disease symptoms. Hence, the high occurrence of the pathogen, across samples is of great concern since analysis of its gene segments showed that it not a vaccine strain. However, an American metagenomic study on the respiratory virome of broilers reported the predominance of *Infectious bronchitis virus* in the normal healthy virome of studied chickens [12]. Also, their occurrence though apathogenic, be attributed to high-rate viral transmission of *Infectious bronchitis virus*/*Avian coronavirus* among birds, particularly in multi-age commercial farm structures. Nevertheless, recent studies have established *Avian coronavirus* as a nephropathogene, causing frequent nasal discharge and conjunctivitis in poultry which could lead to death in chickens and turkeys [59–61].

#### *Caliciviridae*

**Fig. 6:**
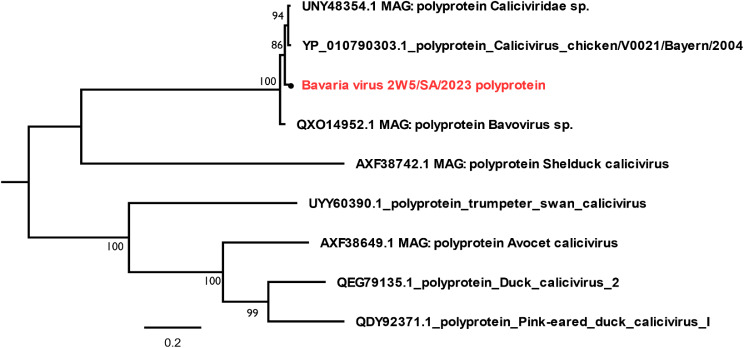
Phylogenetic analysis of the polyprotein gene of *Bavaria virus*, using the maximum likelihood (Jone-Taylor-Thornton model). The trees were midpoint rooted for clarity and the branch length support was estimated using 1000 bootstrap replicates. The virus in red font with black node was identified from chicken faecal sample in this study

The *Calicivirus* identified from this avian study belongs to the only species in the *Bavovirus genera.* This Bavarian virus identified had a total of 12,759 reads (Table S6), with its highest nt sequence coverages of 99.3 and 98.5% emerging from 2S4 and 2S5 samples. The complete CDS, polyprotein and VP2 gene segments of this *Bavaria virus* were retrieved. Further blast analysis using the polyprotein revealed this virus was most similar with 85% nt and 96.1% aa identity to a calicivirus identified from American chickens (KY120883.1). It was thus seen from this study that the *Bavaria virus* often occurred within samples from younger chickens after which they gradually disappeared through 4 weeks, becoming non-existent at 7 weeks. The phylogenetic result of *Bavaria virus* with 99.3% genome coverage from chicken sample 2S4 depicted in Fig. 6 showed that this virus is most similar with the provisional reference sequence (RefSeq) of novel *Calicivirus chicken/V0021/Bayern/2004* (NC_075411.1) virus from Germany (Fig. 6). In addition, it was observed that this virus clustered with the only member of this genus *Bavovirus*. Further alignment of the virus against the non-redundant (nr) NCBI database also showed it shared only 85% nt and 96.1% aa with *Chicken calicivirus strain RS/BR/2015* (KY120883.1) in a different clade in *Nacovirus* genus.

#### *Picornaviridae*

*Picornaviridae* was observed to be the most abundant viral family with a total of 1,550559 viral reads from eight species belonging to five *genera* (Table S5). The species identified in this study includes, *Megrivirus A and C, Gallivirus A, Sicinivirus A, Avisivirus B, Orivirus A, Chicken picornavirus 1 and Quail picornavirus QPV1/HUN/2010.* The prevalence of individual species of the picornaviruses identified in this study is shown in Table 2.

**Table 2:** Prevalence of picornavirus genera in chicken faeces

Family	Picornavirus species identified	Number of incidences (*N* = 27)	Percentage occurrence (%)
*Picornaviridae*	*Sicinivirus A*	27	100.0
	*Gallivirus A*	26	96.3
	*Megrivirus A*	4	14.8
	*Megrivirus C*	20	74.0
	*Avisivirus B*	7	25.9
	*Orivirus A*	14	51.9
	*Chicken picornavirus 1*	24	88.9
	*Quail picornavirus QPV1/HUN/2010*	19	70.4

**Fig. 7:**
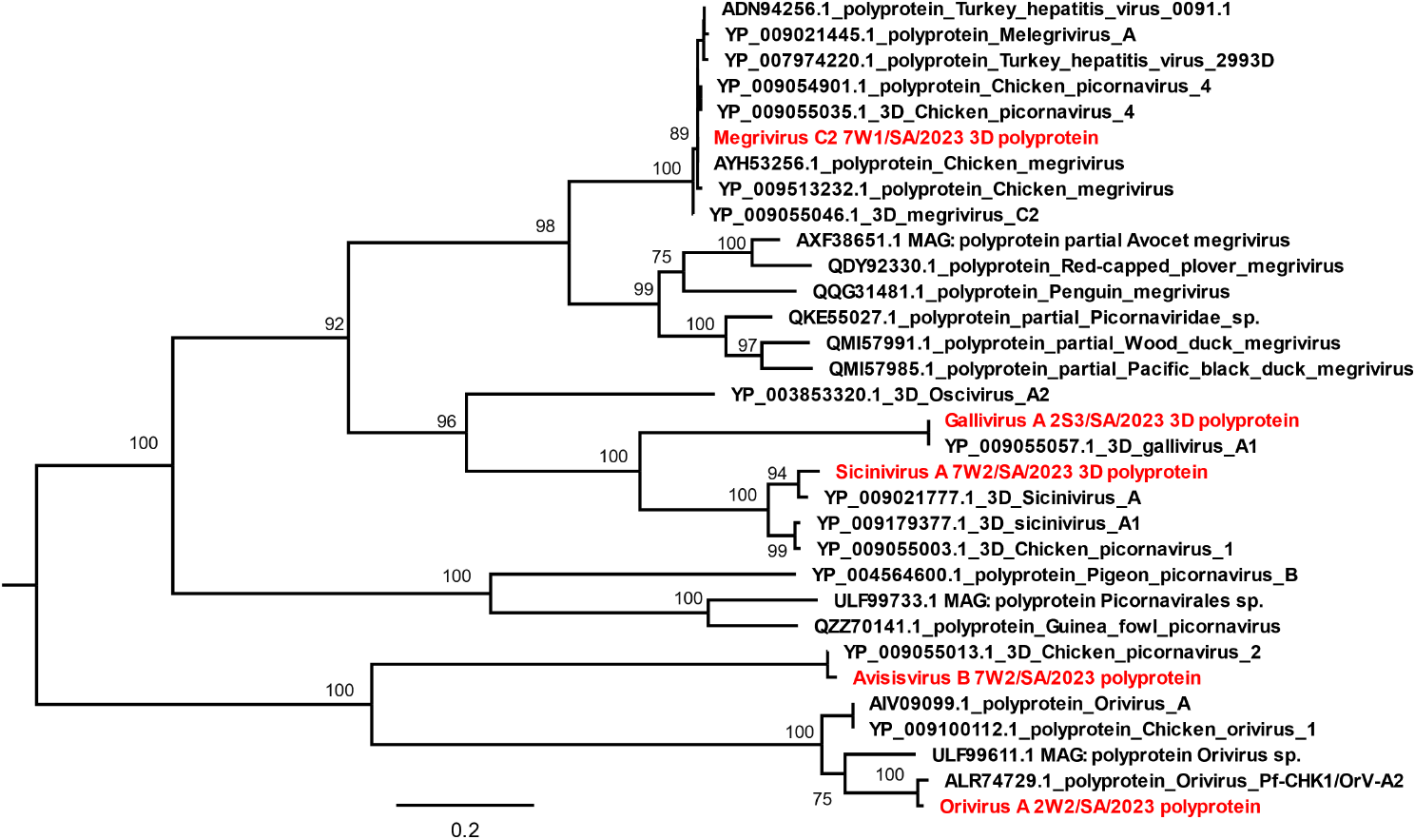
Phylogenetic analysis of members of the *Picornaviridae*, using the maximum likelihood using the polyprotein gene (Jone-Taylor-Thornton model). The trees were midpoint rooted for clarity and the branch length support was estimated using 1000 bootstrap replicates. Viruses in red fonts were identified from chicken faecal samples in this study

The species *Sicinivirus A* had a 100% prevalence across the 27 samples with a total of 195,040 reads (Table S5). Partial nucleotide sequence (96.0%) of *Sicinivirus A* was obtained from sample 7W2 with 144420.35 reads per million (rpm). In addition, nearly complete DF53_gp1 CDS (99.5%), complete RdRp gene crucial for its replication known as 3D^pol,^ alongside the complete sequences of the VP0 to VP3, 2 A, 2B, 2 C, 3 A, 3B and 3 C proteins were recovered. Phylogeny showed its closest relative to be strain UCC001, obtained from chicken cecal samples from Ireland (YP_009021777.1) (Fig. 7). *Megrivirus A* and *Megrivirus C* were obtained with 2303 and 1,121,717 reads, respectively. The results of BLAST alignment at e-value 10^− 3^ of *Megrivirus A* showed its best hits with a *Melegrivirus A* virus conserved in turkey from Hungary (KF961188.1) and a reference sequence of *Turkey hepatitis virus 2993D* at 77.1 and 78.3% identity respectively. In addition, *Gallivirus A* accumulated about 100,247 reads and demonstrated a 96.3% prevalence for picornaviruses (Table 2; Table S6) with highest nt genome coverage of 94.4% from sample 2S3. The complete (100%) protein sequences of 3 A, 3B,3c and 3D (RdRp) genes of *Gallivirus A* was recovered and phylogenetic analysis of its 3D^pol^ showed it identical to 3D of Gallivirus A1 isolate 518 C from Hong Kong (Fig. 7). Furthermore, *Avisivirus B* viral species occurred in 7 samples with highest genome coverage of 92.7% from 2S1. Alignment results of its complete 3D^pol^ RdRp gene showed its best hit of 85.8% identity with *Chicken picornavirus 2 isolate 44 C* from Hong Kong (YP_009055013.1) (Fig. 9). Similarly, *Orivirus A* showed a 51.9% incidence across samples (Table 2) with the highest nt sequence coverage of 96.1% from 2W2 sample in which the complete PI35_gp1 CDS and polyprotein genes was recovered. Phylogenetic results showed that it is most related to the polyprotein gene of strain Pf-CHK1/OrV-A2 from Hungarian chicken (Fig. 7). Additionally, two unclassified avian picornaviruses isolated from this study are *Chicken picornavirus 1* and *Quail picornavirus QPV1/HUN/2010* with 88.9 and 70.4% prevalence across samples, respectively (Table 2). *Chicken picornavirus 1* had very low coverage (59.8%), while *Quail picornavirus QPV1/HUN/2010* was observed to have its highest nt sequence coverage of 94.2% from 4W5 sample whose phylogeny showed close relatedness with Picornavirales *sp.* ULF99733.

#### *Retroviridae*

The two retroviruses obtained from this study were *Avian leukosis virus* and *Rous sarcoma virus*, accruing a total of 4478 and 2201 reads, respectively (Table S6). Although these viruses were identified in very low genome coverage between 36.3 and 63.1%, the two viruses occurred in both summer and winter samples and appeared to have high hit up to 99.6% identity with the same clones *Avian leukosis virus subgroup E* from the UK (MT263508.1) and USA (MF817820.1). Retroviruses causing infections in birds are conserved within the *Alpharetroviru*s. Major cancerous infections occurring in avian species including chickens, have been majorly attributed to these two major retroviruses, *Rous Sarcoma virus* [54] and *Avian leukosis virus* [55, 56]. The presence of these viruses from both summer and winter samples in this study, though at lower coverage show that they may be circulating in commercial poultry farms in South Africa.

#### *Hepeviridae*

*Avian hepatis E virus* or its new name *Avihepevirus magniiecur*, was identified in 10 samples mainly from the winter season yielding a total of 12,126 reads (Table S6). Its near complete genome (98.8%) with complete CDS (BA77_gp1, gp2 and gp3) and non-structural polyprotein was obtained from 7W4. Additional homology comparison with its recovered complete genome revealed that it shared 83.7% homology with a novel divergent isolate 19/03914 *Avihepevirus magniiecur* virus from HSS chickens and pheasants in France (ON922634.1) proposed to belong to genotype 7 [62]. In addition, it also shared 83.66% and 83.68% with isolate W838-14 and W865-14 from liver samples of Australian *Gallus gallus* believed to be a recombinant progeny of US (genotype 2) and Hungarian strain (genotype 4) associated with poultry importations. The identification of this virus in ten chicken samples in both summer and winter periods at high genome coverage (98.8%) suggests a natural subclinical infection of the studied chicken flock. *Avihepevirus magniiecur*, has been numbered as an emerging zoonotic pathogen capable of infecting humans with significant public health concern [63, 64]. Recent studies have identified these aHEV genotypes from different countries to be a major cause of hepatitis-splenomegaly syndrome (HSS) or big liver and spleen disease (BLSD) in chickens [62, 65, 66]. Chickens infected with aHEV may advance from subclinical to rapidly increased mortality and a severe decrease in egg production [67]. Nevertheless, some studies have recovered RNA genomes of aHEV and its antibodies from apparently healthy chickens [65, 68]. Hence, while the detection of aHEV in apparently healthy layer chickens in this study may not have immediate public health implications as a zoonotic pathogen. However, it does raise concerns as to whether HSS occurs more frequently in South Africa, often undiagnosed or unreported. Hence, further studies including different geographic locations in South Africa is needed to determine the genotypes, prevalence, and spread of aHEV in South Africa.

### Identification of non-avian viruses from chicken faeces

The non-avian viruses identified in the current study were mainly food-related viruses, tailed DNA phages, fungal and mammalian viruses with majority of them occurring in very low read abundances (Table S7). The two dsDNA tailed phages identified, *Escherichia virus DE3* and *Lambdavirus lvO276* were both from *Lambdavirus* genera of *Siphoviridae* obtained from 7W3 and 2W4 samples respectively (Table S7). In addition, distinct fungal viruses belonging to two viral families, *Partitiviridae* and *Totiviridae* were found, mainly occurring in low read abundances and in less than three samples, except *Aspergillus fumigatus partitivirus 2* found in 9 samples that encompasses both seasons. Furthermore, diet-associated, and insect-like viruses, *Hubei orthoptera virus 1*, *Hubei picornavirus 24* and *Wuhan insect virus* (Table S7). The occurrence of tail phages *Escherichia virus DE3* and *Lambdavirus lvO276* was not expected due to the DNA depletion and rRNA enrichment steps employed. Nevertheless, previous studies have reported tail phages from families *Myoviridae Podoviridae* and *Siphoviridae* in American [2] and UK [48] chickens, despite the former employing DNA removal procedures. *Aspergillus fumigatus partititivirus* has gained significant attention as a fungal viral pathogen in poultry as an opportunistic pathogen causing immune suppression and respiratory related diseases in chickens [69]. The identification of the fungal virus, *Aspergillus fumigatus partitivirus 2*, is of concern as it is known to cause opportunistic immune related infections in chickens. However, recent studies have also shown that this virus lacks host specificity [69, 70] which may be explained by its presence in the studied chickens. While the identification of food related viruses can be associated with different plant sources of the diet regimens given to chickens, the insect-like viruses may be attributed to use of insects as feed ingredients for poultry. The use of insects seems to have gained attention due to their potential as a sustainable and alternative protein source in animal diets [71]. Previous study by Wille, Harvey [72] identified the *Taggert virus*, a nairovirus from chinstrap penguins, as well as a new *Bruthen virus* from the family *Phenuviridae*, a sister family of *Phlebovirus* that consists of tick association viruses. With the identification of viruses thought to be plant, arthropod and insect- associated from bird’s further research is needed to determine the true host of these viruses and the effect of diet sources on the diversity and abundance of RNA viruses in birds.

The mammalian viruses obtained from the investigated chicken samples includes an unclassified *Picornavirales Tottori-HG1* and non-avian unclassified *Picobirnaviridae* members (Table S7). While *Picornavirales Tottori-HG1 virus*, had a low genome coverage ranging within 38–45%, the non-avian members of *Picobirnaviridae* were moderately abundant across samples with good coverage ranging from 60 to 90%. The identification of diverse mammalian PBVs from more than 10 samples and at high genome coverage in this study is not utterly surprising. The viral family *Picobirnaviridae* lacks host specificity with only one genus *Picobirnavirus* and two genogroups, GI and GII. Currently, there is contradictory information regarding the real host of PBVs, owing to the short sequences used to identify PBVs which are not typical of the whole RdRp gene phylogeny, hence making their defined identification and classification difficult [73]. Not only have PBVs been found in humans, invertebrates, and birds, but they have also been hypothesized to be phages [74, 75] and eukaryotic fungal viruses [76, 77]. Although, the direct and indirect interactions between some of these mammal and poultry cannot be totally ignored. Particularly, humans are responsible for feeding these commercial chickens, dogs are common pets, monkeys are highly abundant in South Africa, and some commercial poultry farms rear other animals such as pig. Recent metagenomic investigation on the Oral RNA virome of backyard swine farms from the KwaZulu-Natal Province, South Africa, identified *Picornavirales Tottori-HG1*, with results showing that it originated from Japan [78] and other studies reported *Picobirnavirus dog/KNA/2015* in non-dog hosts [78, 79] in South Africa. Nevertheless, it remains unclear and difficult to explain how the *Otarine picobirnavirus* occurred in the studied chicken. Notwithstanding, previous studies have identified viruses *Otarine picobirnavirus*, and *Picobirnavirus dog/KNA/2015* in human respiratory samples [79] and swine faecal samples [78] in South Africa. Overall, it is imperative to elucidate underlying the transmission mechanisms and spillover events pertaining to various viruses affecting common farm animals, particularly with a focus on swine viruses, and their spillover and impact on poultry populations.

### Variations in avian RNA viral abundance and diversity as a function of age and sampling season

Analysis of gut RNA virome structure of the faecal samples of chickens in this study revealed differences in diversity and abundances of viral species across the 27 chicken libraries investigated. There was no distinct trend in the dynamics of total viral read across various collated sample time points in the six categories analyzed, as shown in Table S5. A look at the week two summer (2 S) and winter (2 W) groups showed that they had total viral reads of 312,202 and 1,581,024, respectively (Table S5). Hence, there seems to be an observed increase in viral reads between these seasons at week 2. The observed pattern was way different for the 4 weeks chickens which had comparable ranges of total viral reads, which were 256,094 and 224,309, respectively. Another contrasting phenomenon exhibited was in the 7-week samples, showing an opposite effect. It is noteworthy that the 7 W group exhibited a significantly higher number of total viral reads (799,296) in contrast to the 7 S group (29,114), with a nearly 4-fold increase (Table S5). For the two independent sample collection seasons, the findings indicate that during the summer season, there was a negligible reduction of viral reads in the 2 S and 4 S categories. However, a slight increase of viral increase was observed in the 4 S and 7 S chicken groups. Overall, the winter samples exhibited higher number of viral reads.

**Fig. 8:**
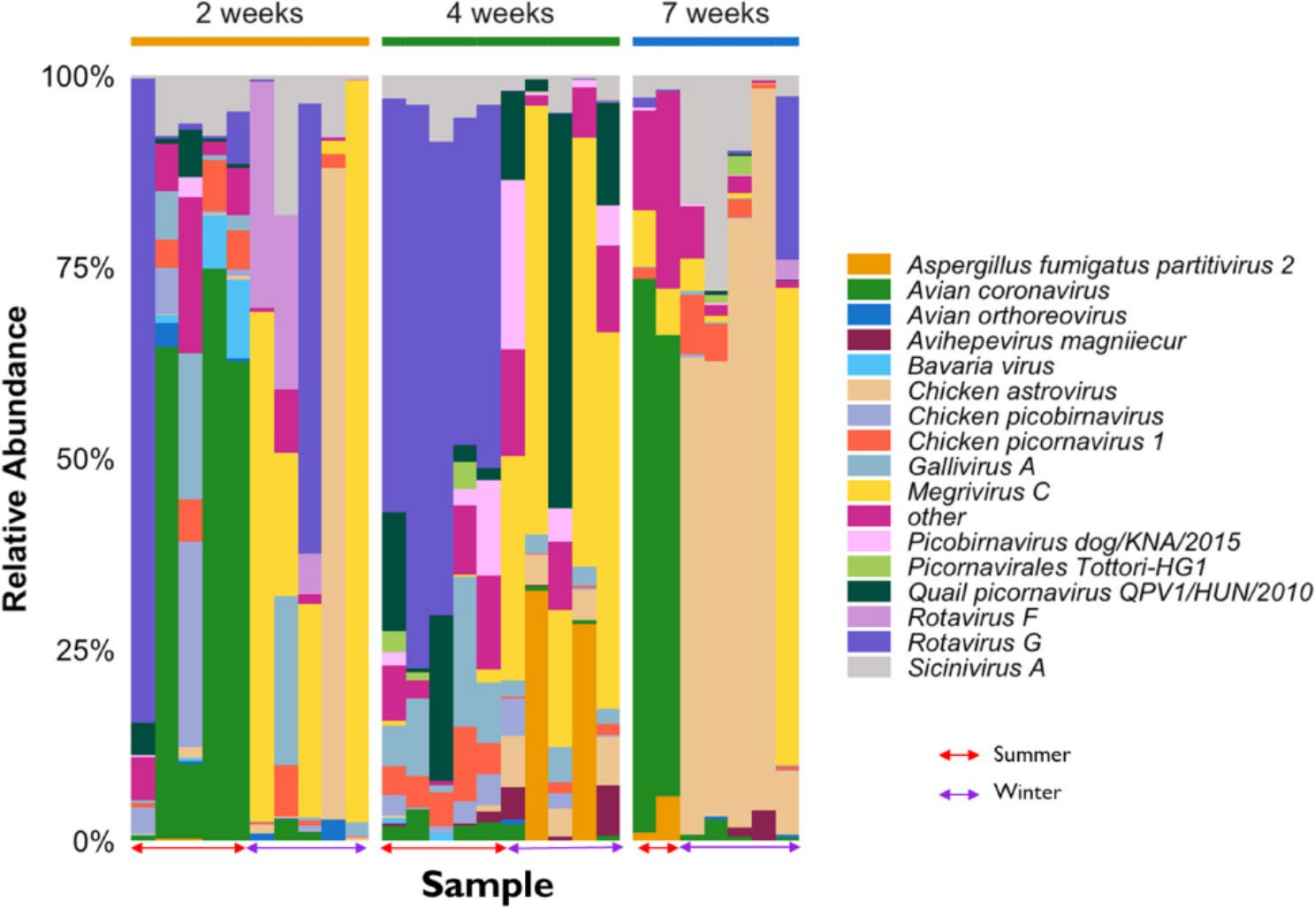
The relative abundance profile of major RNA viral species across the chicken faecal samples at different ages (2, 4 and 7 weeks) and seasons (Summer and winter)

Based on relative standardised abundance, a high proportion of the relative standardised abundance estimates of sequence reads across all the libraries were classed as RNA viruses and the results of their relative abundance from individual sample and group are presented in Fig. 8. The results showed that the most relatively abundant viral species reads across age groups are *Avian coronavirus, Rotavirus G. Chicken astrovirus, Megrivirus C* and *Sicinivirus A* (Fig. 8). In addition, *Bavaria virus, Chicken picobirnavirus Chicken picornavirus 1* and *Aspergillius fumigatus partitivirus 2* were moderately represented. However, the remaining viral species had very low abundance in individual samples, including those assigned in the other group as shown in Fig. 8. It was interesting to see the changing dynamics of members of the *Reoviridae*, particularly, *Rotavirus G* and *Avian orthoreovirus*, which decreased with increasing age (Fig. 8). Notably, members of the *Picornaviridae* family were found throughout all age groups and seasons particularly, *Sicinivirus A* (Fig S3B; Fig. 8). The observed prevalence of *Reoviridae* members, namely *Rotavirus G*, and *Avian orthoreovirus* occurring in both winter and summer samples, with high abundance can be ascribed to the heightened vulnerability of juvenile chickens to reoviruses. This susceptibility arises from the ongoing maturation of their immune system, which is characterized by low immunity to infections. Recent studies have reported a high susceptibility rate of rotaviral infections in younger birds [52, 54], , which is a major cause of runting-stunting/malabsorption syndrome resulting in delayed development and lower productivity in chickens. In addition, the observed significant decrease in reoviruses at 7 weeks may be further explained by the stable adaptation features exhibited by these mature chickens with full grown immune systems. The observed prevalence of picornaviruses may imply that major members of this viral family are commensal and hence may form part of the normal flora of the chicken gut virome. Moreover, different studies have reported picornaviruses to be prevalent in both healthy [10, 12, 80] and diseased [11, 48, 52, 54] bird species including chickens.

In addition, it is notable that certain viral families and/or species within the examined chicken samples exhibited unique trends with respect to age and seasonal variations whereby they are detected in summer samples but are entirely absent in winter samples, or conversely. Notably, it was also revealed that the winter sample were mainly characterized by *Chicken astrovirus* and *Rotavirus G*. Interestingly, this observed seasonal distribution exhibited by some viral species, such as *Rotavirus F, genus Lamdavirus, Infectious bursal disease virus, Festuca pratensis almagavirus 1, Fusarium poae virus 1*, and unclassified RNA virus ShiM-2016 may be attributed to their emergence during the winter period. Similarly, the exclusive occurrence of *Tomato mosaic virus, Pepper mild mottle virus*, and some partitiviruses (*Botryotinia fuckeliana partitivirus 1, Cryptosporidium parvum virus 1, Pythium nunn virus 1, Ustilaginoidea virens partitivirus 2*, and *Verticillium dahliae partitivirus 1*) during the summer periods, may either mean that the viruses are more predominant during the summer or may be associated with the diet (milled grains) taken by these chickens which may carry these fungal viruses. Overall, at viral family levels, all viral families occurred throughout the two seasons explored with exception to some non-avian viral families, *Siphoviridae, Amalgaviridae, Partitiviridae, Potyviridae* and *Totiviridae* comprising of fungal and plant viruses. However, at species level viral diversity varied with some viruses occurring in winter period while being entirely absent in summer or vice versa for instance; *Rotavirus F*. In addition, viral abundance varied more with age rather season, where the effect of age chicken may have been higher than the seasonal effect in the abundance of some of these viruses. Nevertheless, their occurrence by mere chance events cannot be entirely ruled out, particularly with viruses that occurred in lower abundance and in < 3 samples. To the best of our knowledge, the present study is the first to explore the effect of season on viral abundance in chickens, particularly from the African continent. Based on available studies as at the time of this study, there was limited information on the effect of season on viral abundance and diversity in domesticated birds. However, a recent Australian study on wild birds (Pacific black ducks, Chestnut teals, Grey teals and Wood ducks) explored the effect of season on the abundance of the viral families *Picornaviridae* and *Parvoviridae* [80]. Findings from their study showed that picornaviruses were mainly found during late autumn to late winter months while parvoviruses were found throughout the year. In addition, Vibin et al. [80], demonstrated that members of these two viral families varied not only in virus composition across species and time but also in their abundances, despite the sharing the same habitat at the times of sampling. Overall, avian viruses appear to be well represented in all chicken faecal samples. In addition, it could be concluded that about 20 RNA virus species were found in more than ten different chicken samples.

### Impact of age and season on the alpha diversity and beta diversity of chicken RNA virome

#### Alpha diversity

**Fig. 9:**
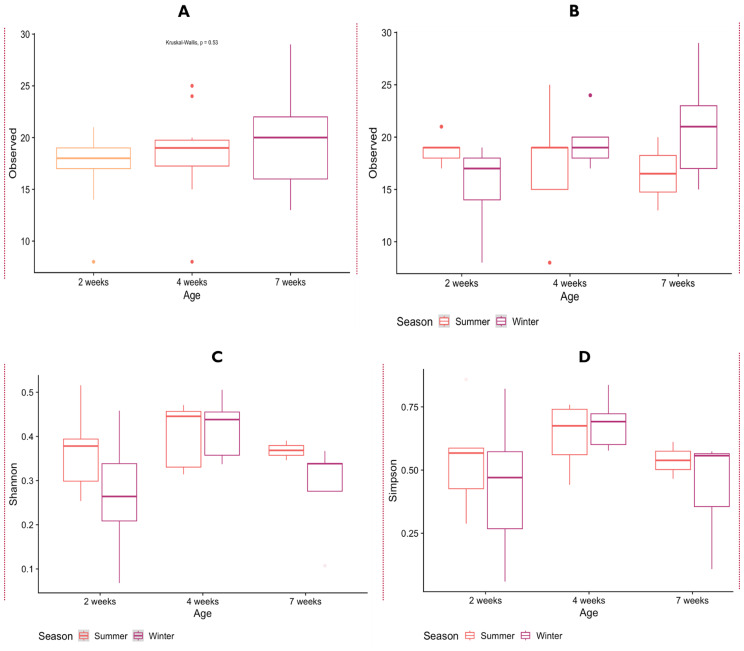
The alpha diversity metric determination of chicken RNA virome as a function of age and season. (**A**) Sobs by age (**B**) Sobs by season (**C**) Shannon-Wiener index, and (**D**) Simpson’s index

The results of the observed alpha diversity principally based on expected count for age and season presented in Fig. 9 showed similar total counts (observed) within ages with interquartile range positively skewed and with most values within the lower quartile (Fig. 9A). For the two seasons, summer and winter, a slight difference was observed at 2 weeks in the within sample counts abundance with the summer sample a bit higher than the winter sample (Fig. 9B). This is not the case with summer and winter samples at week 4, as they demonstrated to have similar viral counts while at week 7, the viral count for the winter sample was higher than that of the summer samples (Fig. 9B). Similarly, the result of alpha diversity (within samples) using metrics that examines both species richness as well as evenness are illustrated in the box plots denoted as Fig. 9C and D. The Shannon’s index considers richness more, while the Simpson’s index places emphasis on the evenness of the viral species. For Shannon diversity, the 4 weeks’ samples had the highest species richness among the three age groups studied (2, 4, 7 weeks). The Shannon’s richness based on season showed that the species dynamics of summer were evidently much higher than winter samples at 2 weeks (Fig. 9C). In contrast to the trend observed at week 2 summer and winter samples, here, the summer and winter samples observed at week 4 appeared to be similar. However, at week 7, a slightly higher species abundance was observed for summer samples than its winter samples. The Simpson’s diversity findings were remarkably comparable to the Shannon’s diversity results, with the most species even samples detected at week 4 for age groups. (Fig. 9D). In addition, the results of diversity measures based on seasons revealed that same species evenness was seen at both summer and winter sample at weeks 4 and 7. However, at week 2, the samples had more species unevenness for their summer and winter collection seasons (Fig. 9D).

With the observed results within sample groups exhibiting similar diversity levels for Shannon and Simpson’s indices, the lack of distinct trend across age groups and seasons can be explained further. First, the viral abundance pattern within each age group can be homogeneous since these chickens are grouped, housed, fed, and vaccinated together, however most commercially bred chickens are multi-age in the same farm. In addition, the inconsistence and higher abundance shown by members of the older chicken at week 7 may be attributed to the lowered immunity of some their members in this group arising from different systemic and recurrent viral infections they may have suffered, leading to different survival mechanism by individual chicken in this age group. This can be explained further by the issue of chance events as has been observed for some transient viral infections, thus leading to chicken individual biased survival features or resistance to some viruses even within the same group. Furthermore, other environmental factors, such as sanitation, lack of biosecurity measures and the introduction of birds from other commercial farms may influence viral abundance.

Overall, the alpha diversity for age (*p* = 0.116; df = 2) and seasons (*p* = 0.172; df = 1) were not significant. Further analysis of variance using the non-parametric Kruskal-Wallis’s H rank sum test on alpha diversity within groups showed that the values for age (*p* = 0.146; R^2^ = 3.849; df = 2) and observed season (*p* = 0.241; R^2^ = 1.371; df = 1) were not significant. A post-hoc analysis, specifically the Bonferroni method and Turkey’s multiple comparisons conducted as a follow up to determine which groups means were not significantly different from each other, showed that the alpha diversity of all individually paired group was not significant (*p* > 0.05). Hence, the observed (*P* > 0.05) no significant effect observed for alpha diversity indicates that regardless of age or seasons, the overall viral abundance of samples within the same group are similar/homogenous. In addition, viral abundance was higher in juvenile chickens (2 weeks) between the three age groups which can be attributed to their immature immune systems resulting in increased susceptibility to infections. Similar no significant alpha diversity result was observed from a recent study that investigated the impact of age in an *Anseriformes* species, *Ruddy turnstones* with P-value > 0.05 [10].

#### Beta diversity

**Fig. 10:**
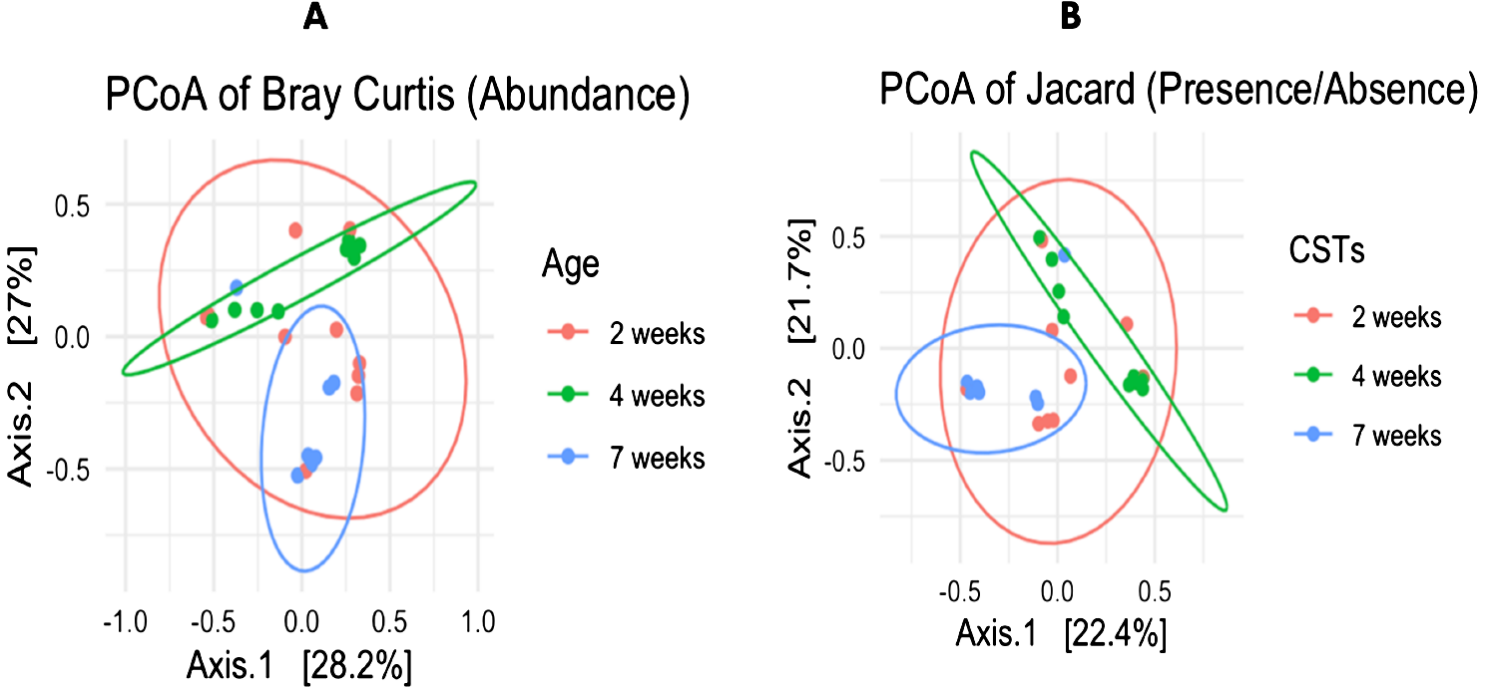
Principal co-ordinate analysis on chicken viral abundance and diversity as functions of age and season, presented as (**A**) Bray-Curtis’s similarity index and (**B**) Jaccard index. The age means the individual age of birds whose samples were obtained while CSTs means the chicken seasonal collection timepoints

The results of the beta diversity metrics for age and season determined using the abundances of the principal coordinate analysis (PCoA) based on the Bray-Curtis and Jaccard showed that with exception of few outliers, the individual samples from weeks 4 and 7 samples clustered distinctively by their ages (Fig. 10A and B). However, for both abundance (Bray-Curtis) and presence/absence (Jaccard) theorems, it was revealed that the 2 weeks samples were more radically spread, clustering with both the weeks 4 and 7 samples (Fig. 10A and B).The observed distinct clustering for both indices (Jaccard and Bray-Curtis) between viruses at week 4 and week 7 may indicate a marked difference in viral diversity between the acclimatization stage (week 4) and the mature stage (week 7) of the chicken. Hence it appears that there is no relationship/ limited connectivity/viral sharing between weeks 4 and 7. However, for week 2, the observed clustering results indicate significant viral connectivity/sharing between week 2 and week 4 as well as week 2 and week 7. Hence it can be deduced that, some of the viruses identified from this chicken at week 2 followed through to week 4 and week 7.

Statistical evaluation of the Bray-Curtis beta diversity metrics showed that the beta diversity between age groups were significant (*P* = 0.01099; R^2^ = 0.17085; df = 2), and consistent with that of the two seasons revealing high significant difference between the seasons (*P* = 0.000999; R^2^ = 0.24107; df = 1). Overall, based on the data sets in this study, the beta diversity (between groups) was revealed to statistically significant for both age and season (*p* < 0.05) and indicates that viral abundance and diversities are greatly influenced by age and season between sample groups for chicken used in this study. Hence it could be deduced that the diversity and abundance between studied chicken samples groups may differ and may be dependent on distinct features characterising each group. Therefore, between group of one or more distinct feature, the diversity and abundance may be heterogenous. Different avian studies have demonstrated marked differences in viral abundance and diversities between sample sets characterized by different features such as age structure, seasonality, host species and latitude [80–82]. Similarly, microbiome studies have attributed bird’s age, feeding pattern/regimens, raising environment and body site to have great impact on the diversity of bacterial population in commercial layers hen [83, 84]. Overall, the differences in diversities and abundance between groups whether age or season, may be attributed to factors such as varying temperature changes for season, different feed formulations in form of diet eaten by these chickens at different ages as well as different vaccines and/or treatment administered to chickens in a specific group which may influence viral abundance and diversities.

## Conclusion

In this study, the faecal RNA virome of asymptomatic South African chickens were analysed at three developmental ages and over two seasons using mNGS. Viral enrichment and purification strategies adopted allowed significant recovery of viruses, including novel chicken astroviruses (CAstV), many previously known avian, mammalian, fungal and plant viruses that may be circulating in poultry farms. While some of these viruses identified were presumed to have commensal roles such as picornaviruses or may be opportunistic pathogens, viruses like rotaviruses, coronaviruses and *Avihepevirus magniiceur* have established severe pathogenic effects. The diversity and abundance of RNA gut viruses were significantly influenced between chicken groups characterized by different ages. In addition, it could also be concluded from this study findings that season play a role in the occurrence of gut virus(es) in chickens. While it is acknowledged that the study is limited by sample size, the findings have provided baseline information that future studies could build on for more encompassing generalization on the viral diversity of chickens in SA. The result from this study reiterates that despite the lack of discernible clinical manifestations, RNA viruses remain prevalent within the GIT of chickens. Thus, chickens may experience recurrent viral infections that could significantly impact the development of their normal gut virome and overall well-being. Hence, with the intensification of poultry systems, routine surveillance of chicken viruses is imperative to assess the risk of disease outbreaks in poultry farming. In addition, monitoring cross species transmission of RNA viruses will serve as an epidemiological step towards curbing the wild spread of viruses from poultry to mammals or conversely. Overall, the present study provided insights on the diversity of chicken faecal virome and would serve as a reference for future investigations, looking to compare virome of healthy and diseased chickens in South Africa.

### Electronic supplementary material

Below is the link to the electronic supplementary material.

Supplementary Material 1

## Data Availability

All data generated during this study are provided within the manuscript and its supplementary information file. All viral sequences analyzed have been submitted and released by the National Center for Biotechnology GenBank under the accession numbers PP228887-PP228924. Raw sequence data obtained from this study has been deposited in the NCBI Sequence Read Archive (SRA) under the BioProject ID: PRJNA1068849 (SRA accessions; SRR27722602-SRR27722628 and BioSample accessions; SAMN39605134-SAMN39605160).

## Abbreviations

GIT: Gastrointestinal tract
rRNA: Ribosomal ribonucleic acid
NGS: Next-generation sequencing
NetoVIR: Novel enrichment techniques of viromes
GD: Genome detective
VP: Viral protein
NSP: Non-structural protein
CAstV: Chicken astrovirus
aHeV: Avian hepatitis E virus
PBVs: Picobirnaviruses
P: P-value
PCoA: Principal coordinate analysis
