# Supplementary material for: A metagenomic investigation of the faecal RNA virome structure of asymptomatic chickens obtained from a commercial farm in Durban, KwaZulu-Natal province, South Africa
Source: BMC Genomics. 2024 Jun 24;25:629. doi: 10.1186/s12864-024-10517-6 (PMC11194887; doi:10.1186/s12864-024-10517-6)
Supplement: Supplementary file 1 — Supplementary Material 1 [file 12864_2024_10517_MOESM1_ESM.docx]

Supplementary information: A Metagenomic investigation of the faecal RNA virome structure of asymptomatic chickens obtained from a commercial farm in Durban, KwaZulu-Natal province, South Africa.

Vivian C. Nwokorogu ^a^, Santhosh Pillai ^a^, James E. San ^c^, Charlene Pillay ^a^, Martin M. Nyaga ^b^, Saheed Sabiu *^a^.


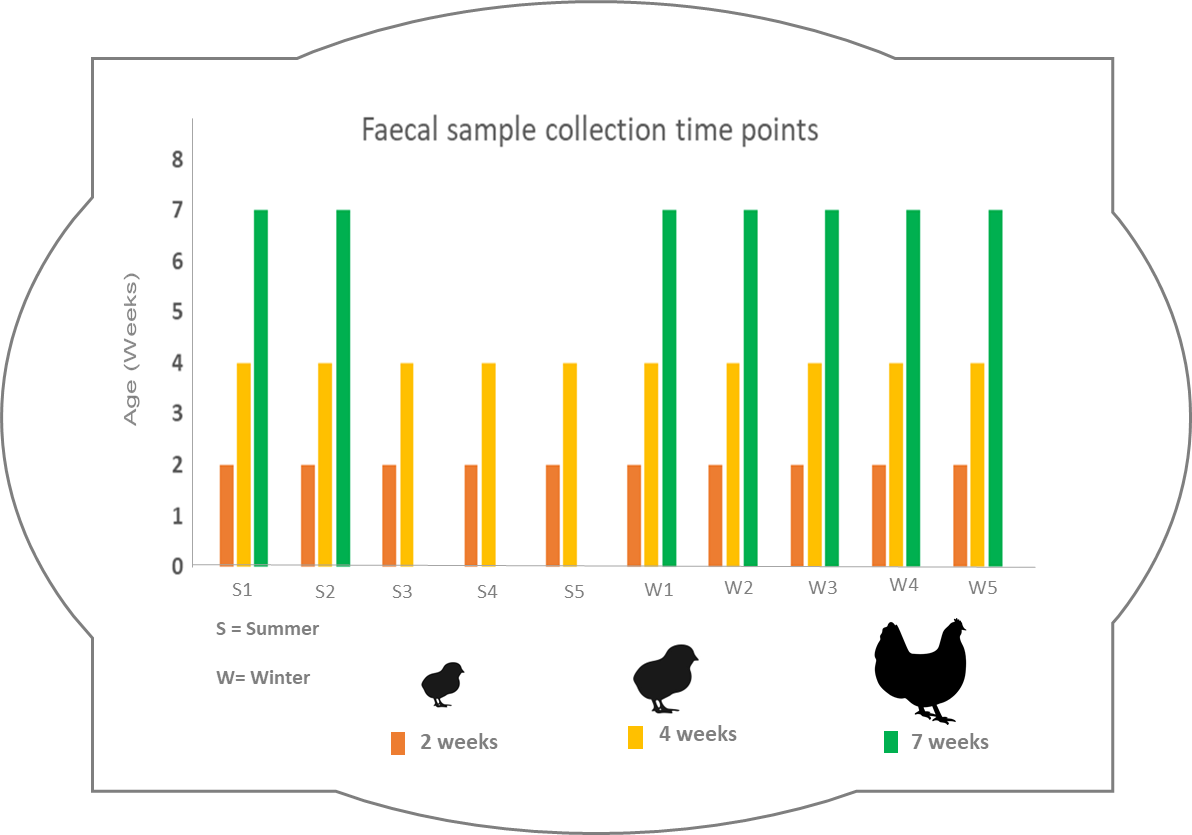


Fig. S1. The time interval of chicken faecal samples collection. A total of 10 samples were collected from 2 weeks and 4 weeks developmental stages, whereas 7 samples were collected only at 7 weeks. On the X-axis, faecal samples S1 to S5 were collected during the summer, whereas samples W1 to W5 were obtained during the winter. In addition, the ages of the chickens used are indicated by the colors of the bars, and each bar represents only one sample.

Table S1. The demographics of the ten different gut RNA virome studied chickens

| **Sample tag** | **Chicken identity (ID)** | **Collection date** | **Collection age (weeks)** | **Sampling**  **Season** |
| --- | --- | --- | --- | --- |
| 2S1 | CH/2022/2S1 | 14/01/2022 | 2 | Summer |
| 2S2 | CH/2022/2S2 | 14/01/2022 | 2 | Summer |
| 2S3 | CH/2022/2S3 | 14/01/2022 | 2 | Summer |
| 2S4 | CH/2022/2S4 | 14/01/2022 | 2 | Summer |
| 2S5 | CH/2022/2S5 | 14/01/2022 | 2 | Summer |
| 4S1 | CH/2022/4S1 | 28/01/2022 | 4 | Summer |
| 4S2 | CH/2022/4S2 | 28/01/2022 | 4 | Summer |
| 4S3 | CH/2022/4S3 | 28/01/2022 | 4 | Summer |
| 4S4 | CH/2022/4S4 | 28/01/2022 | 4 | Summer |
| 4S5 | CH/2022/4S5 | 28/01/2022 | 4 | Summer |
| 7S1 | CH/2022/7S1 | 18/02/2022 | 7 | Summer |
| 7S2 | CH/2022/7S2 | 18/02/2022 | 7 | Summer |
| 2W1 | CH/2021/2W1 | 07/07/2021 | 2 | Winter |
| 2W2 | CH/20212W2 | 07/07/2021 | 2 | Winter |
| 2W3 | CH/2021/2W3 | 07/07/2021 | 2 | Winter |
| 2W4 | CH/2021/2W4 | 07/07/2021 | 2 | Winter |
| 2W5 | CH/2021/2W5 | 07/07/2021 | 2 | Winter |
| 4W1 | CH/2021/4W1 | 21/07/2021 | 4 | Winter |
| 4W2 | CH/2021/4W2 | 21/07/2021 | 4 | Winter |
| 4W3 | CH/2021/4W3 | 21/07/2021 | 4 | Winter |
| 4W4 | CH/2021/4W4 | 21/07/2021 | 4 | Winter |
| 4W5 | CH/2021/4W5 | 21/07/2021 | 4 | Winter |
| 7W1 | CH/2021/7W1 | 11/08/2021 | 7 | Winter |
| 7W2 | CH/2021/7W2 | 11/08/2021 | 7 | Winter |
| 7W3 | CH/2021/7W3 | 11/08/2021 | 7 | Winter |
| 7W4 | CH/2021/7W4 | 11/08/2021 | 7 | Winter |
| 7W5 | CH/2021/7W5 | 11/08/2021 | 7 | Winter |

**Table S2. Details of negative control incorporated in this study**

| Negative controls | Reagents |
| --- | --- |
| Control 1 | Phosphate buffer saline + antibiotics reagents + nuclease enzymes reagents |
| Control 2 | All reagents in QIAamp viral RNA mini kit including the elution buffer |
| Control 3 | All reagents in NEBNEXT Globin rRNA depletion kit |
| Control 4 | All reagents in QIASeq FX Single Cell RNA Library Preparation Kit |

In this study, from the results of the no template controls (Negative controls), the taxa "Rotavirus A", "Rotavirus B", "Bat rotavirus", and "Duck astrovirus GII.A were excluded from downstream analysis. The taxa "Rotavirus A", "Rotavirus B" were detected in the negative control libraries of reagent mix incorporated at different stages, thus being presumed to have originated from contamination that is most likely linked to laboratory reagents. Duck astrovirus GII.A was believed to be contaminants due to index-hopping from another library.

**Vaccination record of the studied chickens**

The documented vaccination records of the sampled chickens from the veterinary expert of the poultry were obtained. The studied chickens received vaccination for Infectious bursal disease virus (Nobilis® Gumboro D78), Infectious bronchitis virus 4-91 serotype (Nobilis® IB 4-91), Marek’s disease virus Nobilis® Rismavac + CA126), and Newcastle disease virus (NDV) (Ranikhet disease vaccine 'F1' strain (RDVF1) and a booster dose of Nobilis® Newcavac) only, mainly administered through intranasal or intramuscular method.

**Table S3: Summarized quantitative metadata enrichment result of all sample used in the current study**

| **Chicken identity (ID)** | **Sample ID** | **Quantification after RNA extraction (ng/µl)** | **RNA quantification after coloum purification post DNase treatment**  **(concentrated to 14 µl)**  **(ng/µl)** | **RNA Quality 260/280 ratio after coloum purification post DNase treatment** | **RNA concentration (ng/µl) post rRNA removal** | **Concentration of cDNA after 10x dilution (ng/ml)** | **Confirmed concentration of normalized cDNA range in 10 µl (ng/µl)** | **Concentration of indexed cDNA after library construction (ng/µl)** |
| --- | --- | --- | --- | --- | --- | --- | --- | --- |
| CH/2022/2S1 | 2S1 | 1.12 | 3.90 | 2.09 | 1.30 | 52500 | 833.9 | 5.38 |
| CH/2022/2S2 | 2S2 | Too low | Too low | 1.98 | Too low | 49100 | 869.8 | 6.94 |
| CH/2022/2S3 | 2S3 | 0.90 | 3.22 | 2.00 | 1.10 | 50700 | 900.0 | 6.58 |
| CH/2022/2S4 | 2S4 | Too low | 0.30 | 2.00 | Too low | 42800 | 889.1 | 12.60 |
| CH/2022/2S5 | 2S5 | Too low | Too low | 1.99 | Too low | 42000 | 947.8 | 15.80 |
| CH/2022/4S1 | 4S1 | Too low | 1.05 | 2.01 | 0.35 | 50100 | 898.0 | 17.80 |
| CH/2022/4S2 | 4S2 | 0.70 | 1.90 | 2.20 | 0.50 | 45600 | 989.1 | 19.20 |
| CH/2022/4S3 | 4S3 | Too low | Too low | 2.06 | Too low | 48500 | 959.9 | 8.44 |
| CH/2022/4S4 | 4S4 | Too low | Too low | 2.00 | Too low | 51300 | 805.8 | 9.06 |
| CH/2022/4S5 | 4S5 | 2.00 | 3.09 | 2.10 | 1.01 | 52400 | 864.3 | 10.80 |
| CH/2022/7S1 | 7S1 | Too low | 6.25 | 2.20 | 2.1 | 35300 | 957.5 | 10.10 |
| CH/2022/7S2 | 7S2 | 4.86 | Too low | 2.01 | Too low | 23800 | 966.4 | 14.90 |
| CH/2021/4W1 | 2W1 | Too low | 0.29 | 2.30 | Too low | 24600 | 988.7 | 12.70 |
| CH/2021/4W2 | 2W2 | 5.95 | 7.49 | 2.19 | 3.03 | 51600 | 898.2 | 9.84 |
| CH/2021/4W3 | 2W3 | 6.40 | 10.70 | 2.03 | 3.59 | 51200 | 943.9 | 11.00 |
| CH/2021/4W4 | 2W4 | 6.30 | 9.04 | 2.00 | 4.01 | 51400 | 968.2 | 22.00 |
| CH/2021/4W5 | 2W5 | 9.06 | 11.27 | 2.01 | 3.85 | 49600 | 911.0 | 12.10 |
| CH/2021/7W1 | 4W1 | 0.80 | 3.2 | 1.99 | 1.10 | 44400 | 936.3 | 13.30 |
| CH/2021/7W2 | 4W2 | 3.30 | 4.02 | 2.06 | 1.34 | 52400 | 890.0 | 12.60 |
| CH/2021/7W3 | 4W3 | Too low | 0.28 | 2.03 | Too low | 41500 | 986.4 | 11.20 |
| CH/2021/7W4 | 4W4 | 5.4 | 6.32 | 2.00 | 2.21 | 36800 | 917.5 | 8.92 |
| CH/2021/2W1 | 4W5 | Too low | 1.02 | 2.02 | 0.45 | 51300 | 940.9 | 5.48 |
| CH/2021/2W2 | 7W1 | 17.40 | 21.29 | 2.00 | 11.40 | 51700 | 954.2 | 9.92 |
| CH/2021/2W3 | 7W2 | Too low | 0.98 | 2.13 | Too low | 49000 | 957.8 | 8.94 |
| CH/2021/2W4 | 7W3 | 16.19 | 19.40 | 1.99 | 7.56 | 51200 | 980.1 | 14.10 |
| CH/2021/2W5 | 7W4 | Too low | 0.85 | 2.10 | Too low | 51800 | 872.0 | 9.82 |
| CH/2021/7W5 | 7W5 | 4.20 | 5.33 | 1.97 | 2.17 | 43800 | 987.6 | 18.60 |
| Control/PBS/A/E | C1 | Too low | Too low | - | Too low | 13100 | 869.8 | 13.0 |
| Control/extraction | C2 | - | Too low | - | Too low | 3900 | 903.9 | 8.96 |
| Control/rRNA | C3 | - | Too low | - | Too low | 1560 | 818.6 | 9.64 |
| Control/cDNA/WTA | C4 | - | - | - | - | 11300 | 801.4 | 3.14 |

**Table S4: Quantitative data of the 27 individual chicken libraries sequenced in this study**

| **Chicken identity (ID)** | **Reads generated per library** | **Contigs generated per library** | **Total viral reads *denovo* assembled** |
| --- | --- | --- | --- |
| 2S1 | 248188 | 220 | 139100 |
| 2S2 | 191852 | 164 | 58040 |
| 2S3 | 404388 | 196 | 91676 |
| 2S4 | 259734 | 146 | 76070 |
| 2S5 | 203588 | 154 | 79954 |
| 4S1 | 267858 | 232 | 79626 |
| 4S2 | 269918 | 155 | 193842 |
| 4S3 | 366620 | 94 | 4360 |
| 4S4 | 46764 | 156 | 13760 |
| 4S5 | 409918 | 198 | 55332 |
| 7S1 | 369820 | 95 | 69860 |
| 7S2 | 150194 | 113 | 13952 |
| 2W1 | 548982 | 124 | 485488 |
| 2W2 | 353808 | 150 | 227142 |
| 2W3 | 340180 | 174 | 189768 |
| 2W4 | 290180 | 135 | 244666 |
| 2W5 | 560630 | 75 | 503054 |
| 4W1 | 427372 | 218 | 61694 |
| 4W2 | 489704 | 181 | 139266 |
| 4W3 | 515600 | 122 | 60166 |
| 4W4 | 231416 | 141 | 23144 |
| 4W5 | 545476 | 139 | 47332 |
| 7W1 | 410972 | 182 | 46024 |
| 7W2 | 512602 | 207 | 300592 |
| 7W3 | 401104 | 240 | 190176 |
| 7W4 | 282050 | 143 | 232626 |
| 7W5 | 330358 | 174 | 242928 |

**Table S5:** Taxonomic classification of chicken viral contigs from faeces based on their respective collection time points

| **Sample groups ID** |  | **2S** | **4S** | **7S** | **2W** | **4W** | **7W** | **Sum of reads** | **Sum of identified viral genera** |
| --- | --- | --- | --- | --- | --- | --- | --- | --- | --- |
| **Collection time (weeks)** |  | **2** | **4** | **7** | **2** | **4** | **7** |  | **21 classified genera and unclassified viruses** |
| **Assigned RNA virus species** | **Viral families** |  |  |  |  |  |  |  | ***Genera*** |
| ***Avian coronavirus*** | ***Coronaviridae* (167789)** | 119890 | 8599 | 20639 | 8321 | 1505 | 8835 | 167789 | *Gammacoronavirus* |
| ***Avisivirus B*** | ***Picornaviridae***  **(1,550559)** | 64 | 108 | 0 | 0 | 0 | 2412 | 2584 | *Avisivirus* |
| ***Chicken picornavirus 1*** |  | 11510 | 10599 | 340 | 22473 | 1116 | 20084 | 66122 | *Unclassified Picornaviridae* |
| ***Gallivirus A*** |  | 9488 | 23493 | 43 | 60002 | 6062 | 1159 | 100247 | *Gallivirus* |
| ***Megrivirus A*** |  | 0 | 0 | 1687 | 0 | 338 | 278 | 2303 | *Megrivirus* |
| ***Megrivirus C*** |  | 0 | 765 | 2098 | 868617 | 106007 | 144230 | 1121717 |  |
| ***Orivirus A*** |  | 1653 | 1890 | 0 | 17752 | 0 | 728 | 22023 | *Orivirus* |
| ***Quail picornavirus QPV1/HUN/2010*** |  | 6994 | 9660 | 0 | 152 | 24512 | 1789 | 43107 | *Unclassified Picornaviridae* |
| ***Sicinivirus A*** |  | 13665 | 9620 | 794 | 71793 | 3483 | 95685 | 195040 | *Sicinivirus* |
| ***Chicken astrovirus*** | ***Astroviridae* (666244)** | 1103 | 431 | 0 | 210819 | 9789 | 444105 | 666244 | *Avastrovirus* |
| ***Bavaria virus*** | ***Caliciviridae* (12759)** | 12188 | 371 | 0 | 200 | 0 | 0 | 12759 | *Bavovirus* |
| ***Avian leukosis virus*** | ***Retroviridae***  **(6679)** | 1334 | 921 | 272 | 0 | 345 | 1606 | 4478 | *Alpharetrovirus* |
| ***Rous sarcoma virus*** |  | 726 | 359 | 132 | 13 | 165 | 806 | 2201 |  |
| ***Avihepevirus magniiecur*** | ***Hepeviridae***  **(12126)** | 0 | 619 | 0 | 122 | 3046 | 8339 | 12126 | *Avihepevirus* |
| ***Avian orthoreovirus*** | ***Reoviridae***  **(632918)** | 1358 | 19 | 0 | 11269 | 136 | 1664 | 14446 | *Orthoreovirus* |
| ***Rotavirus F*** |  | 0 | 0 | 0 | 199481 | 156 | 5813 | 205450 | *Rotavirus* |
| ***Rotavirus G*** |  | 93322 | 169238 | 334 | 101470 | 90 | 48568 | 413022 |  |
| ***Infectious bursal disease virus*** | ***Birnaviridae* (16)** | 0 | 0 | 0 | 0 | 0 | 16 | 16 | *Avibirnavirus* |
| ***Chicken picobirnavirus*** | ***Picobirnaviridae***  **(75633)** | 13648 | 3186 | 0 | 1264 | 1879 | 654 | 20631 | Unclassified *Picobirnavirus* |
| ***Orthopicobirnavirus hominis*** |  | 889 | 753 | 8 | 227 | 2751 | 311 | 4939 | *Orthopicobirnavirus* |
| ***Otarine picobirnavirus*** |  | 7321 | 3001 | 5 | 4099 | 2403 | 1027 | 17856 | *Unclassified Picobirnavirus* |
| ***Picobirnavirus dog/KNA/2015*** |  | 1124 | 4472 | 132 | 439 | 7951 | 969 | 15087 |  |
| ***Picobirnavirus green monkey/KNA/2015*** |  | 1845 | 802 | 0 | 724 | 1519 | 321 | 5211 |  |
| ***Picobirnavirus sp.*** |  | 351 | 932 | 0 | 651 | 1055 | 686 | 3675 |  |
| ***Porcine picobirnavirus*** |  | 2972 | 2766 | 0 | 644 | 1294 | 558 | 8234 |  |
| ***Escherichia virus DE3*** | ***Siphoviridae* (1766)** | 0 | 0 | 0 | 0 | 0 | 634 | 634 | *Lambdavirus* |
| ***Lambdavirus lvO276*** |  | 0 | 0 | 640 | 492 | 0 | 0 | 1132 |  |
| ***Aspergillus fumigatus partitivirus 2*** | ***Partitiviridae***  **(50671)** | 240 | 17 | 497 | 0 | 46788 | 15 | 47521 | *Gammapartitivirus* |
| ***Botryotinia fuckeliana partitivirus 1*** |  | 0 | 0 | 7 | 0 | 0 | 0 | 7 | *Unclassified Partitiviridae* |
| ***Cryptosporidium parvum virus 1*** |  | 1009 | 0 | 0 | 0 | 0 | 0 | 1009 | *Cryspovirus* |
| ***Fusarium poae virus 1*** |  | 0 | 0 | 0 | 0 | 9 | 0 | 9 | *Betapartitivirus* |
| ***Penicillium aurantiogriseum partitivirus 1*** |  | 0 | 0 | 6 | 0 | 0 | 0 | 6 | *Unclassified Partitiviridae* |
| ***Penicillium aurantiogriseum partiti-like virus*** |  | 0 | 6 | 0 | 0 | 0 | 816 | 822 | *Unclassified Partitiviridae* |
| ***Penicillium stoloniferum virus F*** |  | 0 | 0 | 99 | 0 | 30 | 0 | 129 | *Gammapartitivirus* |
| ***Pythium nunn virus 1*** |  | 0 | 0 | 334 | 0 | 0 | 0 | 334 | *Unclassified Partitiviridae* |
| ***Sclerotinia sclerotiorum partitivirus S*** |  | 0 | 0 | 0 | 0 | 809 | 0 | 809 | *Unclassified Partitiviridae* |
| ***Ustilaginoidea virens partitivirus 2*** |  | 0 | 0 | 13 | 0 | 0 | 0 | 13 | *Unclassified Partitiviridae* |
| ***Verticillium dahliae partitivirus 1*** |  | 0 | 0 | 12 | 0 | 0 | 0 | 12 | *Unclassified Partitiviridae* |
| ***Festuca pratensis amalgavirus 1*** | ***Amalgaviridae* (18)** | 0 | 0 | 0 | 0 | 0 | 18 | 18 | *Unclassified Amalgaviridae* |
| ***Tomato mosaic virus*** | ***Potyviridae* (7)** | 0 | 0 | 7 | 0 | 0 | 0 | 7 | *Potyvirus* |
| ***Pepper mild mottle virus*** | ***Virgaviridae* (965)** | 0 | 0 | 719 | 0 | 0 | 0 | 719 | *Tobamovirus* |
| ***Tobacco mild green mosaic virus*** |  | 0 | 0 | 14 | 0 | 0 | 232 | 246 |  |
| ***Eimeria tenella RNA virus 1*** | ***Totiviridae* (9724)** | 347 | 0 | 0 | 0 | 0 | 180 | 527 | Unclassified *Victorivirus* |
| ***Scheffersomyces segobiensis virus L*** |  | 9197 | 0 | 0 | 0 | 0 | 0 | 9197 | *Totivirus* |
| ***Picornavirales Tottori-HG1*** | **Unclassified Picornavirales**  **(9894)** | 0 | 3467 | 0 | 0 | 258 | 6169 | 9894 | Unclassified *Picornavirales* |
| ***Hubei orthoptera virus 1*** | **Unclassified viruses**  **(1687)** | 0 | 0 | 0 | 0 | 0 | 906 | 906 | **Unclassified RNA virus ShiM-2016** |
| ***Hubei picorna-like virus 24*** |  | 0 | 0 | 282 | 0 | 0 | 398 | 680 |  |
| ***Wuhan insect virus 22*** |  | 0 | 0 | 0 | 0 | 0 | 101 | 101 |  |
| **Total faecal viruses** | **Viral families** | **Total group viral reads** | | | | | | **Total reads** |  |
| **48** | **15** | 312202 | 256094 | 29114 | 1581024 | 224309 | 799296 | 3202039 |  |

The brown highlight indicates the age group with the highest viral read abundance for each virus presented in rows, while the ash colour tone means that a specific virus(es) is entirely absent in that group(s). In addition, values enclosed in brackets for viral families equates the total number of reads for each viral family.


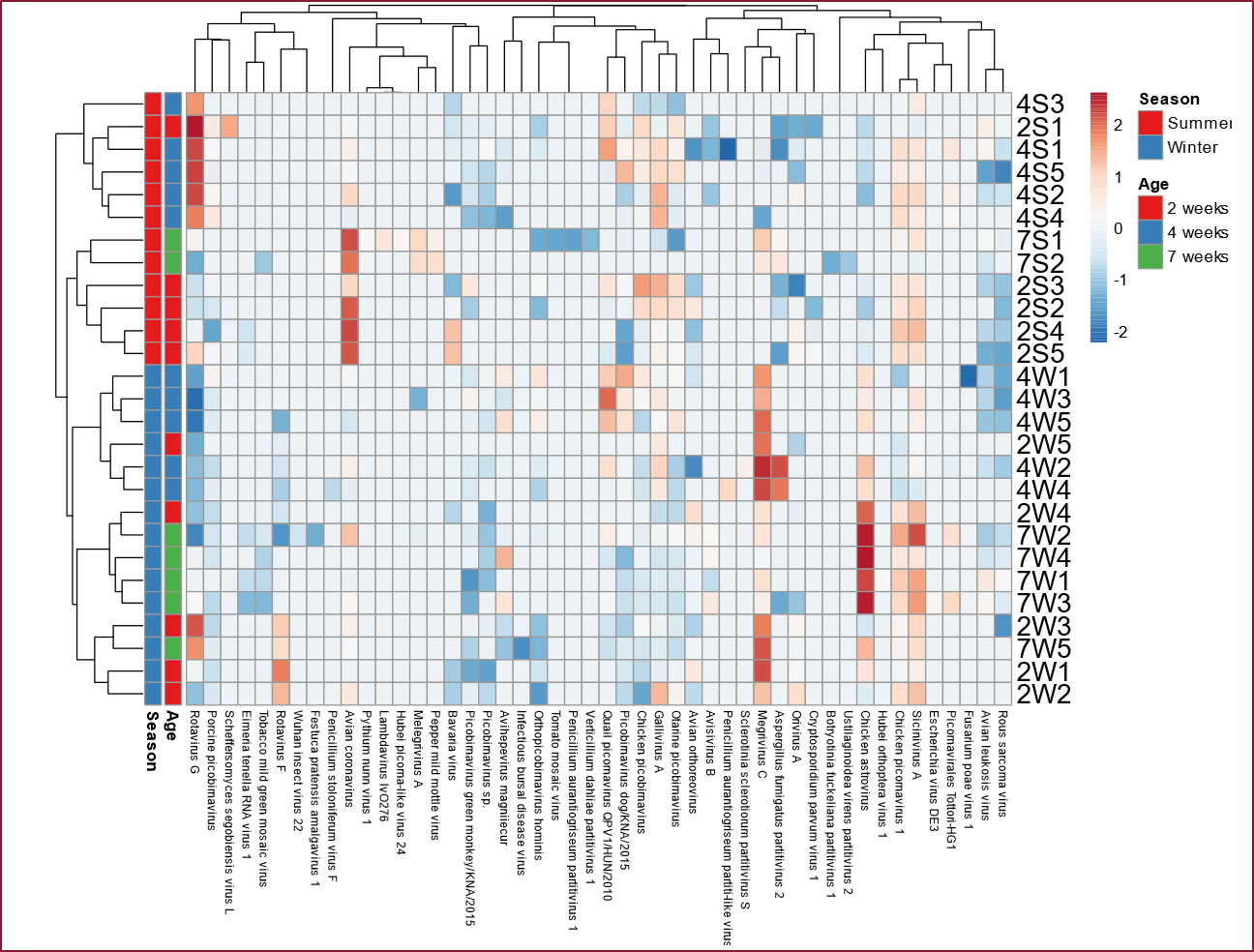


**Fig. S2** The overall read abundance of viral species across individual 27 samples categorized by summer and winter sample collection time points.


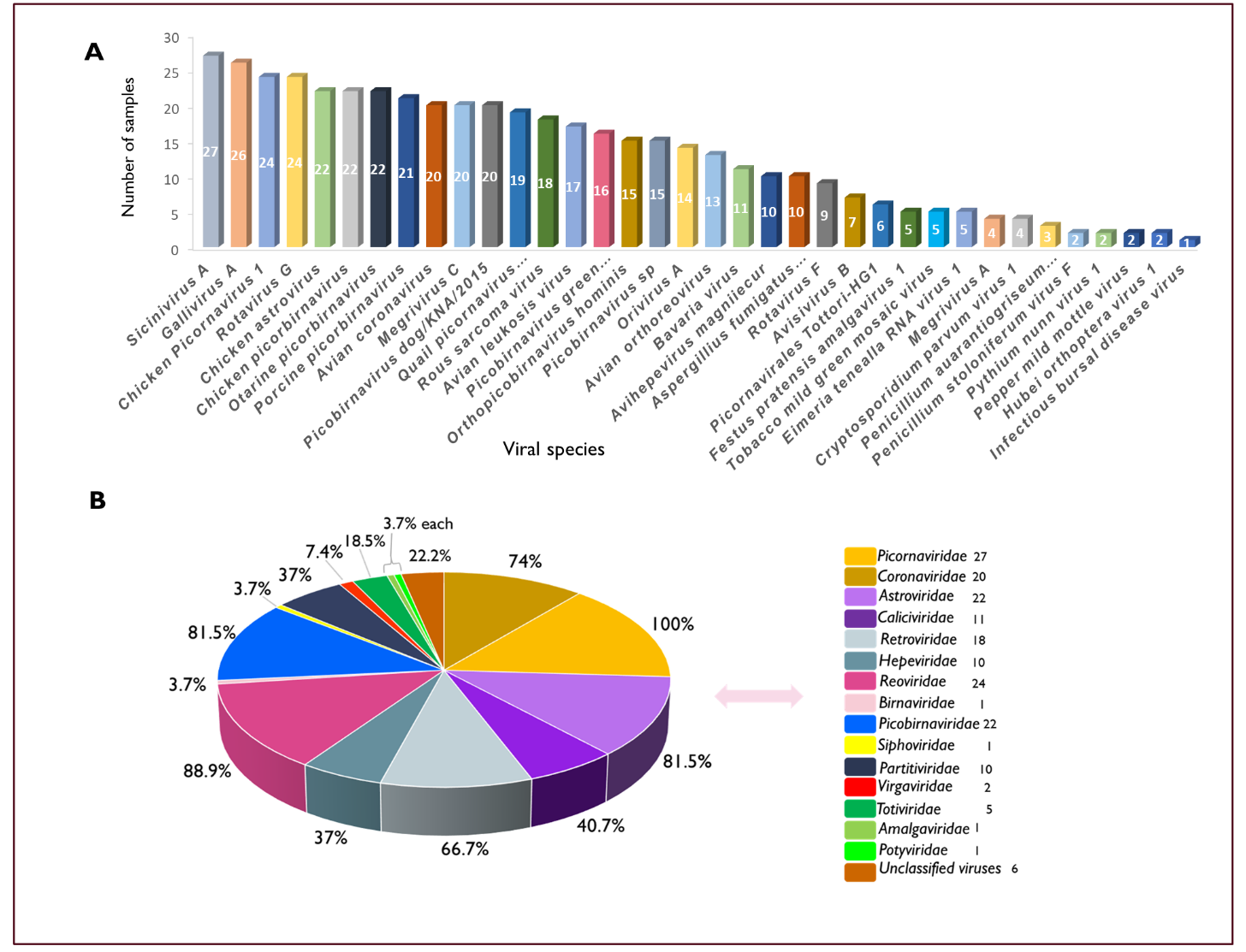


**Fig. S3** Occurrence rate of viral families and species across the 27 studied chicken samples. **A.** rate of identification individual viral species. The values centrally placed in each bar depicts the total number of samples where each corresponding virus was identified. **B.** Pie chart of percentage incidence of the identification of each viral family across samples. The color legend on the right corresponds to each family in the pie chart.

Table S6 Taxonomic classification of avian RNA viruses identified from chicken faecal samples

| **No of avian viral species** | **Genome type** |  |  | **Total viral reads** | **Genome coverage** | **Sum of identified avian viral genera** |
| --- | --- | --- | --- | --- | --- | --- |
|  |  |  |  |  |  | **15 genera + unclassified viruses** |
| **Avian RNA viral species** |  |  | **Viral families** |  |  | ***Genera*** |
| ***Avian coronavirus*** | **Single-stranded RNA**  **(2418740 reads)** |  | ***Coronaviridae*** | 167789 | 94.7-99.3% | *Gammacoronavirus* |
| ***Avisivirus B*** |  |  | ***Picornaviridae***  **(1550559 reads)** | 2584 | 60.3-92.7% | *Avisivirus* |
| ***Chicken picornavirus 1*** |  |  |  | 66122 | 50-59.8% | *Unclassified Picornaviridae* |
| ***Gallivirus A*** |  |  |  | 100247 | 80.9-94.4% | *Gallivirus* |
| ***Megrivirus A*** |  |  |  | 2303 | 84.5-93.1% | *Megrivirus* |
| ***Megrivirus C*** |  |  |  | 1121717 | 90-100% |  |
| ***Orivirus A*** |  |  |  | 22023 | 89.5-96.1% | *Orivirus* |
| ***Quail picornavirus QPV1/HUN/2010*** |  |  |  | 43107 | 81.5-94.2% | *Unclassified Picornaviridae* |
| ***Sicinivirus A*** |  |  |  | 195040 | 87.7-96.0% | *Sicinivirus* |
| ***Chicken astrovirus*** |  |  | ***Astroviridae*** | 666244 | 94-100% | *Avastrovirus* |
| ***Bavaria virus*** |  |  | ***Caliciviridae*** | 12759 | 98-99% | *Bavovirus* |
| ***Avian leukosis virus*** |  |  | ***Retroviridae***  **(6679)** | 4478 | 36.3-63.2% | *Alpharetrovirus* |
| ***Rous sarcoma virus*** |  |  |  | 2201 | 38.2-59.7% |  |
| ***Avihepevirus magniiecur*** |  |  | ***Hepeviridae*** | 12126 | 80.3-98.8% | *Avihepevirus* |
| ***Avian orthoreovirus*** | **Double-stranded RNA**  **(710283 reads)** |  | ***Reoviridae***  **(632918)** | 14446 | 83-99% | *Orthoreovirus* |
| ***Rotavirus F*** |  |  |  | 205450 | 93.4-100% | *Rotavirus* |
| ***Rotavirus G*** |  |  |  | 413022 | 98.3-100% |  |
| ***Infectious bursal disease virus*** |  |  | ***Birnaviridae*** | 16 | 23% | *Avibirnavirus* |
| ***Chicken picobirnavirus*** |  |  | ***Picobirnaviridae*** | 20631 | 92.3-99.9% | Unclassified *Picobirnavirus* |
| ***Scheffersomyces segobiensis virus L*** |  |  | ***Totiviridae*** | 9197 | 45.5% | *Totivirus* |
| ***Aspergillus fumigatus partitivirus 2*** |  |  | ***Partitiviridae*** | 47521 | 85.5-97.8% | *Gammapartitivirus* |
| **Total avian viruses** |  |  | **Avian viral families** |  |  |  |
| **21** |  |  | **11** | 3129023 |  | **15 genera + unclassified viruses** |


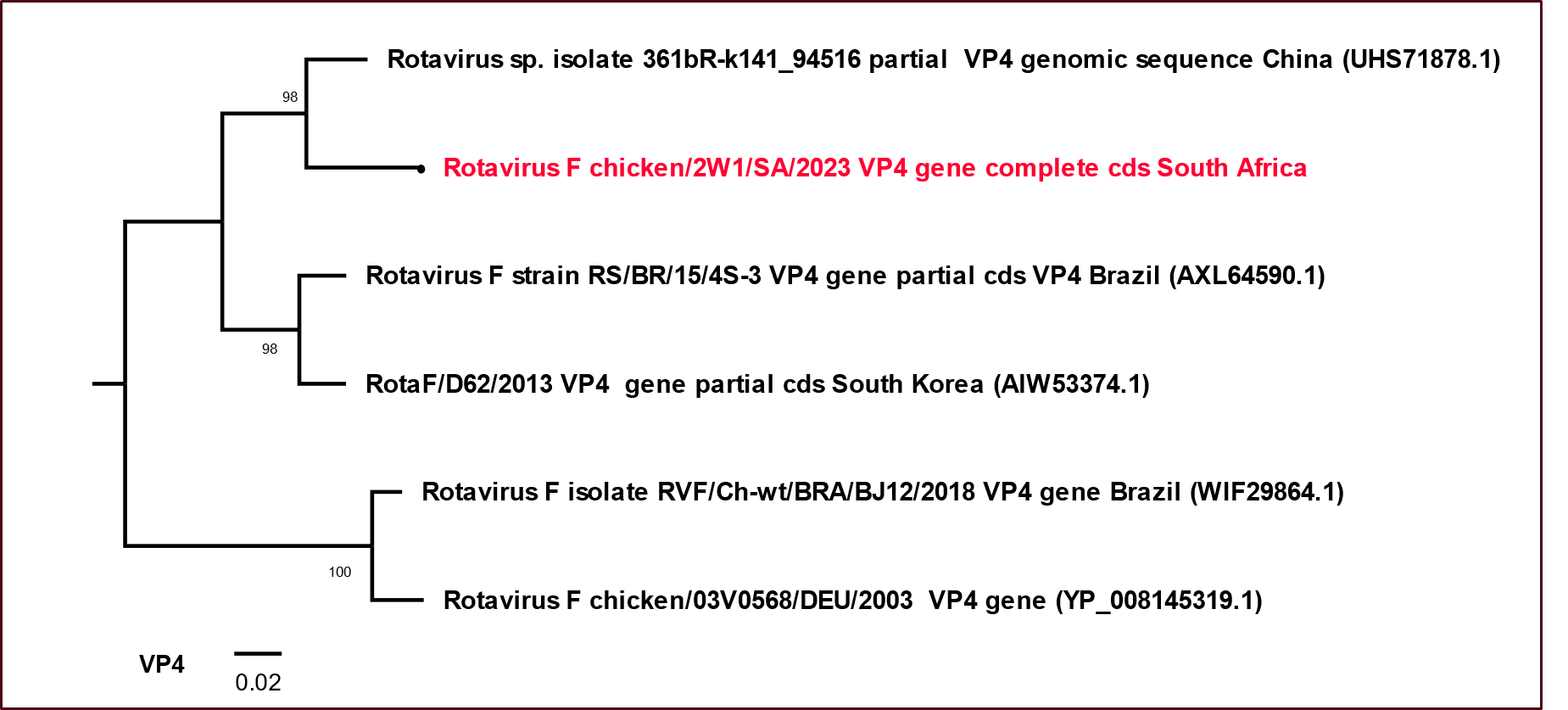


Fig S4. Phylogenetic analysis of *Rotavirus F* virus VP4 segment, using the maximum likelihood, Tamura 3 parameter. The tree was midpoint rooted for clarity and the branch length support was estimated using 1000 bootstrap replicates. Viruses were annotated by their strain name while the virus label in red fonts with red triangle was identified from chicken faecal sample in this study. Virus in blue font are strains from Brazil while those in purple are from South Korea and China.


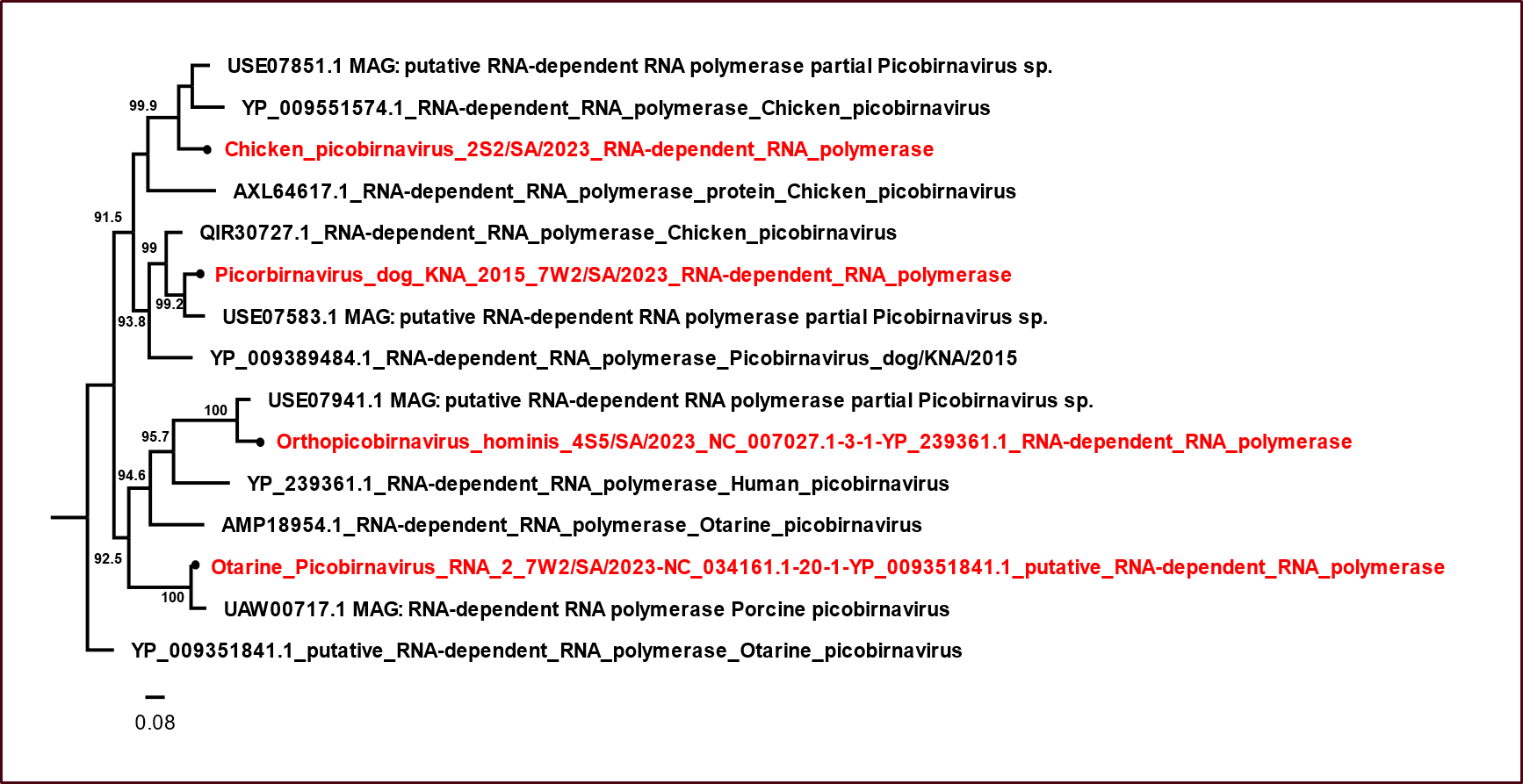


Fig S5. The maximum likelihood phylogenetic analysis of Picobirnaviridae species using the RdRp gene. The tree was midpoint rooted for clarity and the branch length support was estimated using 1000 bootstrap replicates with the Tamura Jone-Taylor-Thornton model. Viruses identified in this study are depicted in red fonts.


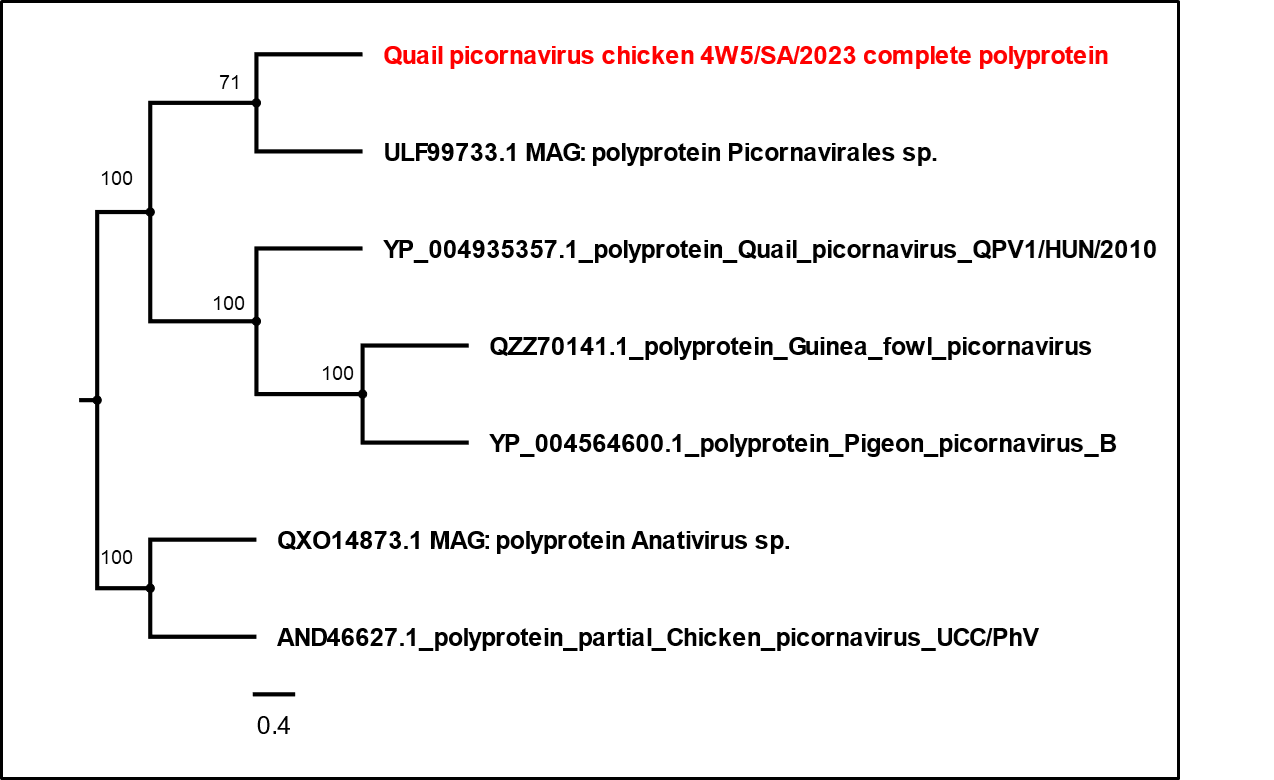


Fig S6. Phylogenetic analysis of Quail picornavirus QPV1/HUN/2010 from sample 4W5, using the maximum likelihood using the polyprotein gene (Jone-Taylor-Thornton model). The tree was midpoint rooted for clarity and the branch length support was estimated using 1000 bootstrap replicates. Viruses in red fonts were identified from chicken faecal sample in this study.

Table S7 Non-avian viruses identified from chicken faecal samples

| **No of avian viral species** | **Genome type** | **Host** | **Viral families** | **Sum of reads** |  | **Sum of identified viral genera** |
| --- | --- | --- | --- | --- | --- | --- |
|  |  |  |  |  |  |  |
| **Non-avian RNA virus species** |  |  |  |  |  | ***Genera*** |
| ***Orthopicobirnavirus hominis*** | ***Double-stranded RNA*** | **Mammalian viruses** | ***Picobirnaviridae***  **(332)** | 4939 |  | *Orthopicobirnavirus* |
| ***Otarine picobirnavirus*** |  |  |  | 17856 |  | *Unclassified Picobirnavirus* |
| ***Picobirnavirus dog/KNA/2015*** |  |  |  | 15087 |  |  |
| ***Picobirnavirus green monkey/KNA/2015*** |  |  |  | 5211 |  |  |
| ***Picobirnavirus sp.*** |  |  |  | 3675 |  |  |
| ***Porcine picobirnavirus*** |  |  |  | 8234 |  |  |
| ***Picornavirales Tottori-HG1*** | **Single-stranded RNA** |  | **Unclassified virus** | 9894 |  | Unclassified *Picornavirales* |
| ***Escherichia virus DE3*** | ***Double-stranded DNA*** | **Phages** | ***Siphoviridae*** | 634 |  | *Lambdavirus* |
| ***Lambdavirus lvO276*** |  |  |  | 1132 |  |  |
| ***Botryotinia fuckeliana partitivirus 1*** |  | **Fungal viruses** | ***Partitiviridae*** | 7 |  | *Unclassified Partitiviridae* |
| ***Cryptosporidium parvum virus 1*** |  |  |  | 1009 |  | *Cryspovirus* |
| ***Fusarium poae virus 1*** |  |  |  | 9 |  | *Betapartitivirus* |
| ***Penicillium aurantiogriseum partitivirus 1*** |  |  |  | 6 |  | *Unclassified Partitiviridae* |
| ***Penicillium aurantiogriseum partiti-like virus*** |  | **Fungal viruses** |  | 822 |  | *Unclassified Partitiviridae* |
| ***Penicillium stoloniferum virus F*** |  |  |  | 129 |  | *Gammapartitivirus* |
| ***Pythium nunn virus 1*** |  |  |  | 334 |  | *Unclassified Partitiviridae* |
| ***Sclerotinia sclerotiorum partitivirus S*** |  |  |  | 809 |  | *Unclassified Partitiviridae* |
| ***Ustilaginoidea virens partitivirus 2*** |  |  |  | 13 |  | *Unclassified Partitiviridae* |
| ***Verticillium dahliae partitivirus 1*** |  |  |  | 12 |  | *Unclassified Partitiviridae* |
| ***Festuca pratensis amalgavirus 1*** | **Double-stranded RNA** | **Diet associated viruses** | ***Amalgaviridae*** | 18 |  | *Unclassified Amalgaviridae* |
| ***Tomato mosaic virus*** | **Single-stranded RNA** |  | ***Potyviridae*** | 7 |  | *Potyvirus* |
| ***Pepper mild mottle virus*** |  |  | ***Virgaviridae*** | 719 |  | *Tobamovirus* |
| ***Tobacco mild green mosaic virus*** |  |  |  | 246 |  |  |
| ***Eimeria tenella RNA virus 1*** | **Double-stranded RNA** |  | ***Totiviridae*** | 527 |  | Unclassified *Victorivirus* |
| ***Hubei orthoptera virus 1*** |  |  | **Unclassified viruses** | 906 |  | Unclassified RNA virus ShiM-2016 |
| ***Hubei picorna-like virus 24*** |  |  |  | 680 |  |  |
| ***Wuhan insect virus 22*** |  |  |  | 101 |  |  |
| **Total enteric viruses** |  |  | **Viral families** | **Total reads** |  |  |
| **27** |  |  | **7** | **73016** |  | **8** |
